# Advanced wound healing with Stimuli-Responsive nanozymes: mechanisms, design and applications

**DOI:** 10.1186/s12951-025-03558-w

**Published:** 2025-07-01

**Authors:** Xiaoyang Liu, Huihui Zhang, Lianglong Chen, Zesen Zheng, Wenwen Li, Chaoyang Huang, Hai Zhou, Yanqi Chen, Ziwei Jiang, Jiaqi Liang, Qiuyi Yu, Lei Yang

**Affiliations:** Department of Burns, Nanfang Hospital, Southern Medical University, Guangzhou, 510515 China

**Keywords:** Diabetic foot ulcers, Chronic wounds, Biomimetic catalysis, Smart therapeutics

## Abstract

Wound healing outcomes critically depend on precise regulation of oxidative and antimicrobial microenvironments. Traditional dressings have limited wound responsiveness, insufficient infection control, and limited treatment accuracy. In contrast, nanozymes, featuring enzyme-mimetic activities, tunable catalysis, and engineered sizes that balance catalytic site accessibility with tissue penetration, offer spatiotemporal control of reactive oxygen species (ROS) and pathogen elimination. This review systematically examines recent advances in stimuli-responsive nanozymes for wound management, focusing on their catalytic mechanisms and therapeutic specificity. These intelligent systems dynamically adapt catalytic behaviors (e.g., ROS scavenging, bacterial lysis) to physical stimuli (temperature, light, ultrasound) and physiological signals (pH, redox imbalance, ATP levels, microbial metabolites), leveraging size-dependent targeting mechanisms to ensure localized therapeutic effects while minimizing off-target damage. Current evidence demonstrates their multifunctional capacity to synergistically accelerate infection clearance, inflammation resolution, and angiogenesis. Future development should prioritize biosafety validation alongside size-effect standardization, stimulus specificity, and scalable manufacturing to advance personalized nanomedicine for refractory wounds.

## Graphical abstract

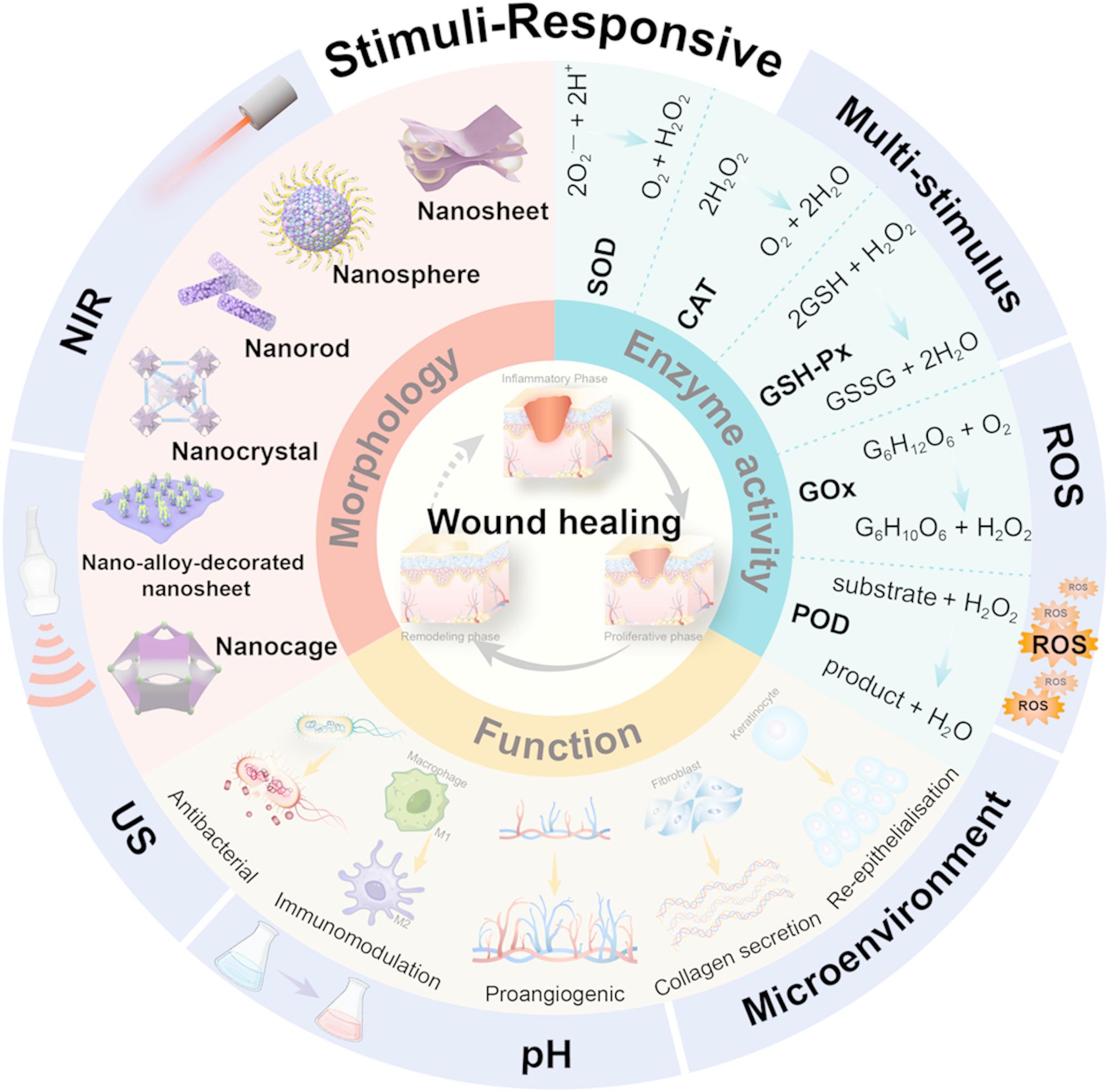

## Introduction

**Fig. 1:**
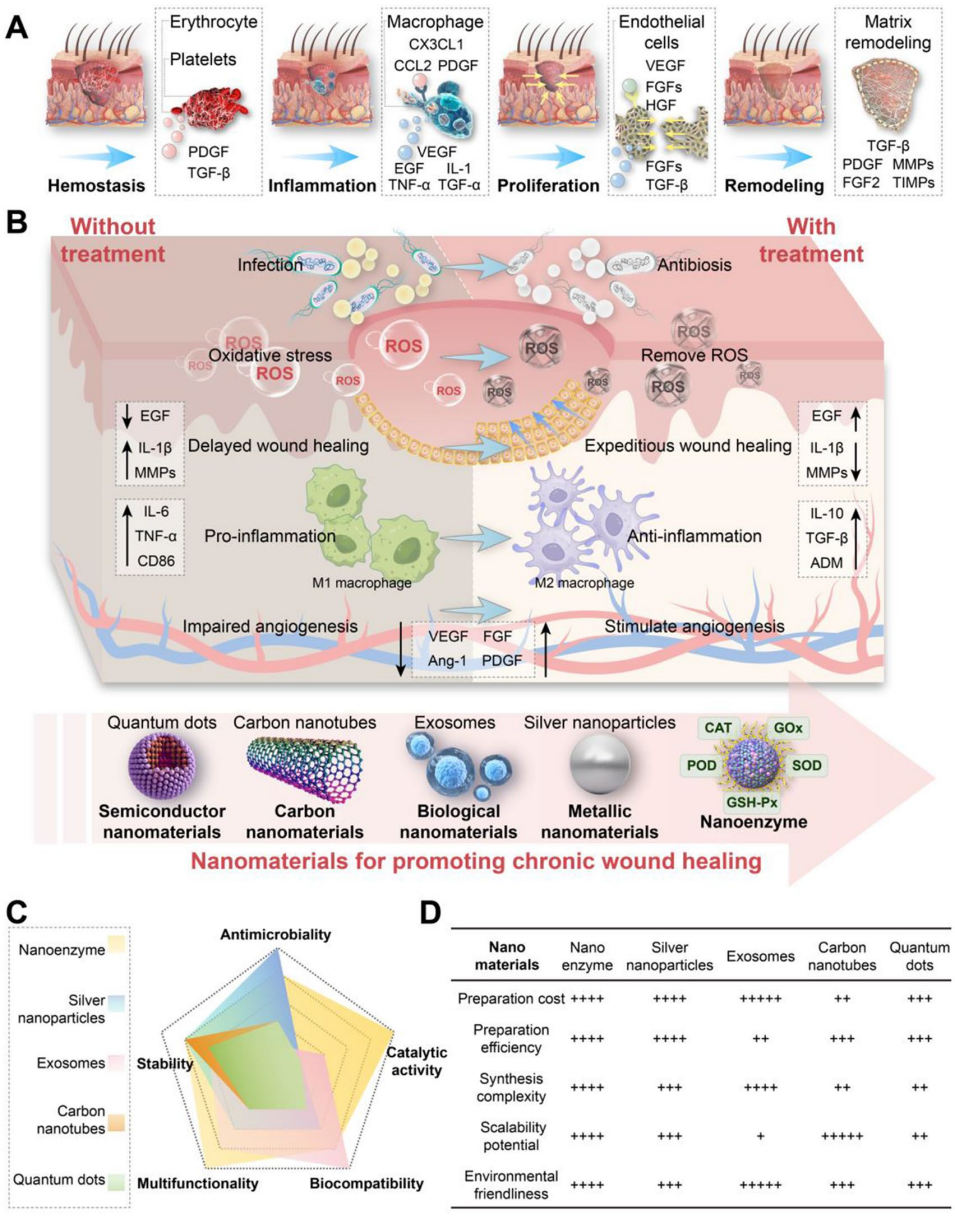
Wound healing stages and nanomaterial applications. **(A)** Schematic diagram of normal wound healing stages: hemostasis, inflammation, proliferation, and remodeling. **(B)** Comparison of chronic wound healing processes between untreated and nanomaterial-treated conditions. Untreated wounds exhibit infection, oxidative stress, and impaired healing, while nanomaterial-treated wounds show reduced infection and oxidative stress with enhanced angiogenesis and anti-inflammatory responses to accelerate healing. **(C)** Radar chart comparing key attributes of nanomaterials (nanozymes, silver nanoparticles, exosomes, carbon nanotubes, and quantum dots) in chronic wound healing: antimicrobial activity, stability, multifunctionality, biocompatibility, and catalytic activity. **(D)** Table summarizing the preparation cost, efficiency, synthesis complexity, scalability potential, and environmental friendliness

The skin acts as a protective barrier and is vital for fluid and electrolyte balance [ 1, 2 ]. However, it can be damaged by surgery and physical trauma, impairing its functions and metabolism [ 3 ]. Upon skin injury, the body immediately initiates a repair mechanism to restore the integrity of the skin barrier. The process of skin wound healing is divided into four coordinated and sequential stages: hemostasis, inflammation, proliferation, and remodeling (Fig.  1 A) [ 4 ]. However, the process of wound healing is often not perfectly orderly; excessive inflammation and bacteria-breeding infection may lead to abnormal skin wound repair and prolong the healing time [ 5 ]. Damage to the skin provides an optimal opportunity for bacterial invasion, particularly infections caused by *Staphylococcus aureus* ( *S. aureus* ), which can lead to severe tissue damage [ 6 ]. Wound management, especially for chronic wounds such as diabetic foot ulcers (DFU), pressure ulcers, and arterial ulcers, has emerged as a significant challenge in healthcare worldwide [ 7 ]. In the United States alone, DFUs account for over 70,000 annual lower limb amputations, generating direct medical costs of $25 billion [ 8 ]. High-income countries have significant health challenges, but low- and middle-income regions face even greater issues. In Nigeria, 18.7% of people have DFUs [ 9 ]. The country also struggles with poor healthcare infrastructure, a lack of specialized wound care services, and inadequate diabetes education. As a result, amputation rates in Nigeria are much higher than in developed nations [ 10 ]. As chronic wounds become more common worldwide, it is very important to develop effective treatment plans [ 11 ].

Wound dressings are of critical significance in the wound healing process. They are fabricated to cover the wound region, arrest bleeding, construct a protective barricade, preclude contamination, sustain appropriate moisture levels, and curtail the loss of body fluids and proteins [12]. However, traditional wound dressings, mainly made of gauze and band-aids, lack the biological properties needed for effective skin repair. They tend to stick to the wound, and changing them often can cause secondary skin damage during removal, leading to discomfort and pain for patients, which hinders healing [13, 14]. To this end, various biomaterials, including electrospun scaffolds [15], foams [16], sponges [17], and hydrogels [18], have been designed to accelerate wound healing and improve patients’ quality of life. Despite their potential, these methods have limitations, including a lack of dynamic response and low drug release efficiency [19]. With the advancement of nanotechnology, nanomedicine (e.g., nanozymes, metallic nanomaterials [20], bio-derived nanomaterials [21], carbon-based nanomaterials [22], semiconductor nanomaterials [23], etc.) has witnessed rapid development over the past several decades and has demonstrated significant potential as a promising therapeutic approach. Nanomaterials, due to their unique physicochemical properties such as high specific surface area, tunable surface activity, and targeted delivery capabilities, are widely integrated into wound dressings [24]. Nanomaterial-enhanced dressings impart multifunctionality to traditional dressings, including antibacterial and anti-infection properties, promotion of angiogenesis and tissue regeneration, and inflammation regulation [25]. Nanomedicine is advancing wound dressings towards dynamic response and real-time monitoring by combining precise diagnosis with treatment strategies [26].

Nanozymes, as a prominent example of nanomaterials, exhibit intrinsic enzyme-like activities that efficiently catalyze substrates into the desired products. Owing to the integration of nanomaterials and natural enzymes, which present unique advantages, a variety of nanozymes based on diverse materials, such as carbon nanomaterials, metal organic frameworks (MOFs), metal oxide nanoparticles (NPs), noble metal NPs, and so on, have been developed at a rapid pace and have been widely utilized in biomedical applications [27, 28]. These enzyme-mimetic nanomaterials provide significant advantages over natural enzymes. They exhibit enhanced catalytic activity across a broad range of pH and temperature conditions. Additionally, they offer greater design flexibility, prolonged blood circulation time, and the capability to integrate multiple enzyme activities effectively [29]. In promoting the healing of chronic wounds, nanozymes exhibit more prominent advantages compared to other nanomaterials, including superior antimicrobial activity, stability, multifunctionality, biocompatibility, and catalytic activity (Fig. 1C). Nanozymes exhibit distinct benefits, such as lower preparation costs, higher preparation efficiency, and simpler synthesis. They also have excellent scalability and are environmentally friendly. As a result, they are considered highly promising materials in nanomedicine (Fig. 1D) [30–35]. These unique properties and multifunctional capabilities make nanozymes stand out as a superior choice to traditional nanomaterials. Nanozyme-based therapeutic systems for wound treatment have attracted substantial attention [36].

Nanozymes possess the unique characteristics of nanomaterials and the biocatalytic functions of natural enzymes such as superoxide dismutase (SOD), catalase (CAT), and glutathione peroxidase (GSH-Px) [37]. They also exhibit excellent reactive oxygen species (ROS) scavenging capacity, high stability under physiological conditions, and rapid clearance in vivo with favorable biocompatibility [38]. Furthermore, nanozymes can catalyze the generation of ROS to eradicate bacteria without inducing drug resistance [39]. For instance, nanozymes exhibiting peroxidase (POD)-like or oxidase (OXD)-like activities have been shown to produce substantial amounts of ROS for effective bacterial elimination [40]. Nanozymes with DNA-like activity present a novel approach to combating drug resistance by targeting and degrading bacterial resistance genes. These nanozymes mimic natural DNA-interacting enzymes, like DNases, allowing them to bind to and cleave DNA sequences responsible for drug resistance [41]. The strategies for the application of nanozymes in promoting diabetic wound healing can be classified into three types: eradicating bacterial infections [42], decreasing blood glucose levels in the diabetic wound [43], and relieving inflammation [44].

Regulating the catalytic activities of nanozymes is a critical concern in biomedical applications [45]. In many instances, nanozymes exhibit continuous catalytic activity, which, in the context of biomedical applications, presents risks to healthy tissues. Such persistent activity could inadvertently cause harm or even substantial damage to normal cellular structures. Consequently, a variety of stimuli-responsive nanozymes have been rationally designed, and their activities can be precisely regulated by multiple stimuli, including but not limited to light irradiation, heat, US, pH, hydrogen peroxide (H_2_O_2_), and glutathione (GSH) [46]. These stimuli-responsive nanozymes activate under specific conditions, minimizing potential damage to surrounding healthy tissues. Moreover, their controlled release not only safeguards the nanozymes in vivo during blood circulation but also facilitates synergistic or cascading actions of different functional components. Due to their precise adjustability, facile functionalization, and biological inertness, responsive nanozymes have been widely applied in biomedical fields.

For a comprehensive discussion of the role of nanozymes in diabetic wound healing, we sourced all relevant publications for our study from the Web of Science Core Collection (WOSCC), which includes over 12,000 high-impact, top-quality scientific journals. Our search employed the following keywords: TS = ((“nanoenzyme” OR “nanozyme”) AND (“stimuli-responsive” OR “responsive” OR “triggered” OR “controlled”) AND (“wound healing” OR “wound” OR “ulcer” OR “dermal” OR “skin” OR “tissue repair”)). We focused on high-impact articles published in recent years, excluding non-English publications, as well as editorial materials, book chapters, conference proceedings, papers, letters, news items, corrections, and other types of content. This strategy ensured that our review was based on the most relevant and quality literature available.

**Fig. 2:**
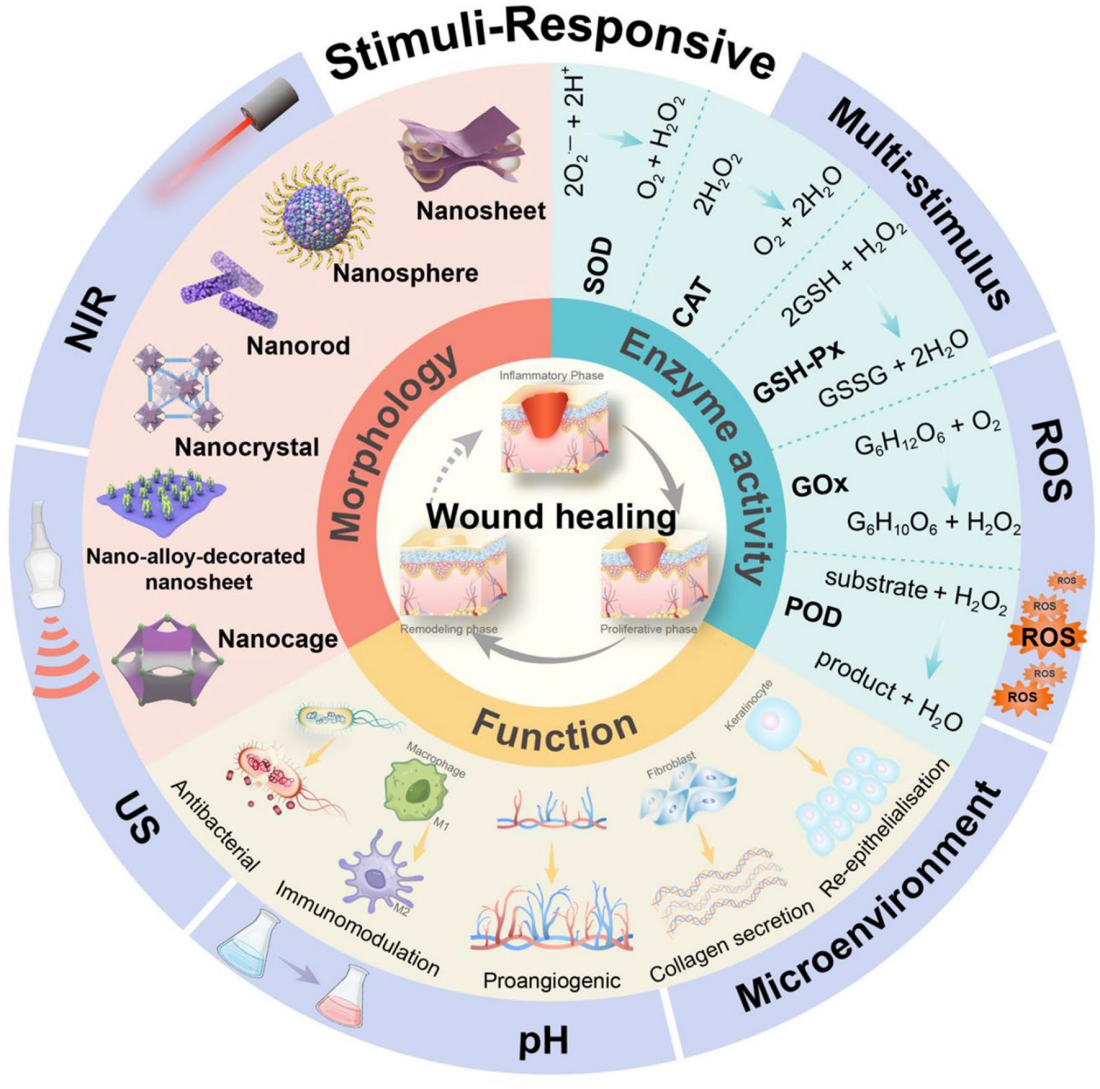
Schematic representation of how stimuli-responsive nanozymes are activated by diverse stimuli to regulate oxidative stress, reduce infections, and promote a balanced environment for efficient wound tissue repair

This review comprehensively analyzes nanozyme applications in wound healing management, with a particular focus on diabetic conditions. Beginning with the fundamental mechanisms of physiological and pathological healing processes, we establish a therapeutic context for nanozyme interventions. The analysis progresses through dual classification frameworks: first examining antioxidant versus pro-oxidant enzymatic functionalities, then systematically exploring stimulus-responsive systems, including physical (temperature/light/US), chemical/biochemical (pH/redox/ATP/microenvironment), and multi-modal responsive nanozymes (Fig. 2). We extensively discuss current translational challenges, including biosafety optimization, specificity enhancement, mechanistic elucidation, and scalable manufacturing. This review aims to present a strategic framework for accelerating the clinical translation of intelligent nanozyme platforms while addressing critical challenges in their adaptation for precision medicine applications.

## Physiological processes and factors in wound healing

### Homeostatic wound repair mechanisms

The skin plays a pivotal role in maintaining homeostasis by mitigating the egress of essential fluids and impeding the ingress of detrimental entities such as deleterious agents and pathogenic organisms [47]. The healing process is initiated immediately after tissue damage, involving a precise, well-orchestrated cascade characterized by the sequential recruitment of diverse cell types in the wound microenvironment. Under normal conditions, the wound healing process comprises 4 distinct but overlapping stages: hemostasis, inflammation, proliferation, and remodeling [48]. It is triggered by blood clotting and the prompt onset of an inflammatory response. Right after the injury, the trabecular vessels constrict to halt bleeding, thereby achieving hemostasis. This process is followed by clot formation involving thrombin and platelets [49]. The inflammatory phase includes the release of chemokines, attraction of immune cells, production of growth factors, promotion of keratinocyte and fibroblast development, and expression of multiple toll-like receptors [50]. The proliferation phase includes keratinocytes, fibroblasts, and endothelial cells. Keratinocytes cause re-epithelialization [51]. In response to signaling molecules, fibroblasts secrete fibronectin and collagen, thereby generating a new permanent extracellular matrix [52]. Endothelial cells proliferate and migrate towards the wound surface to meet metabolic requirements. New blood vessels anastomose with the pre-existing ones to form a stable vascular network. Remodeling represents the final phase of wound healing. At this stage, the intricate vascular system contracts and undergoes thrombosis, resulting in a reduced number of vessels that are more robust and stable [52]. In the remodeling phase, fibroblasts utilize matrix metalloproteinases to substitute type III collagen with type I collagen as the healing process advances [53]. During this process, the collagen protofibrils within the scarred dermis are aligned in a parallel pattern, which is distinct from the original woven configuration of intact skin. Remodeling can take several months to a year, after which wound healing is considered complete [4].

### Dysregulated healing in diabetes

Patients facing issues such as impaired wound healing, metabolic dysregulation within the wound microenvironment, and severe infections experience significant pain, which adversely affects their quality of life. Acute wounds typically progress through an inflammatory phase lasting from a few days to several weeks; in contrast, chronic refractory wounds may necessitate months or even years for complete healing [54]. Normal wound repair follows a structured, sequential process [55]. In contrast, the healing process in DFU wounds is disorganized, with a chaotic progression through these stages, leading to chronic wounds that often fail to heal [56]. Patients with diabetes and chronic wounds like DFUs face a high risk of recurrent infection and potential amputation, significantly increasing morbidity and mortality rates worldwide while imposing substantial healthcare costs [57].

In diabetes, various pathological factors delay or disrupt the wound healing process, which leads to dysfunction and chronicity. Enhanced expression of pro-inflammatory cytokines, excessive oxidative stress, bacterial infections, and biochemical disturbances like hyperglycemia and dyslipidemia contribute to tissue destruction. These factors also promote the development and expansion of the inflammatory microenvironment [58]. This pathological stagnation is mechanistically linked to hyperglycemia-induced metabolic dysregulation [59]. Specifically, hyperglycemia drives excessive ROS production in endothelial cells, which triggers fibronectin/collagen overproduction, non-enzymatic protein glycosylation, and chronic inflammatory signaling [60]. Critically, prolonged poor glycemic control establishes a self-perpetuating cycle: elevated oxidative stress and altered cellular metabolism persist even after glycemic normalization, creating a ‘metabolic memory’ that sustains microcirculatory dysfunction [60]. Increased glucose levels also contribute to the formation of advanced glycation end products, which compromise keratinocytes and fibroblasts by impairing growth factor activity and increasing pro-inflammatory cytokine activity [61]. Furthermore, chronic hyperglycemia-induced glycosylation disrupts intercellular communication in keratinocytes, directly impairing their migratory capacity at the wound edge and perpetuating failed re-epithelialization [62]. This cellular dysfunction synergizes with concurrent vascular impairments that disrupt the normal healing cascade. Specifically, DFU-associated microvascular dysfunction delays the hemostatic phase by reducing blood flow, which diminishes platelet recruitment and growth factor release, thereby slowing fibrin clot formation [63]. The resulting hypoxic microenvironment further compromises tissue oxygenation. Additionally, impaired monocyte trafficking during the inflammatory phase creates a permissive niche for bacterial colonization and biofilm establishment **(**Fig. 1B**)** [64].

### Diabetic microenvironment modulators

The diabetic wound healing microenvironment exhibits pathological complexity, deviating from the classical sequential progression of hemostasis, inflammation, proliferation, and tissue remodeling. Instead, it manifests dysregulated phase overlap and persistent inflammatory activation [65]. Central to this pathology are hypoxia and excessive ROS, which synergistically drive the chronicity of DFUs through two main mechanisms. First, they disrupt inflammatory resolution by impairing macrophage phenotypic transition and suppressing immune cell functionality. This exacerbates necrotic tissue accumulation and creates a pro-infective niche as a result of programmed cell death [66].

Chronic hyperglycemia in individuals with diabetes mellitus induces systemic pathophysiological perturbations that critically impair wound repair. Elevated glucose levels promote the formation of advanced glycation end products, which compromise leukocyte function and dermal structural integrity. Concurrently, hyperglycemia disrupts neovascularization, exacerbating local ischemia and hypoxia—a milieu that accelerates neuropathy and further amplifies inflammatory dysregulation. This metabolic derangement further creates a nutrient-rich environment favoring bacterial proliferation, rendering diabetic ulcers highly susceptible to microbial colonization and secondary infections [67].

DFUs frequently harbor polymicrobial communities, including *S. aureus*, *Escherichia coli*, and *Pseudomonas aeruginosa*, that colonize the wound bed and activate persistent immune-inflammatory responses [68–70]. Bacterial infections perpetuate a cycle of inflammation, directly impeding re-epithelialization and granulation tissue formation [68]. Pathogens within DFUs often organize into biofilms that confer resistance to both host defenses and conventional antibiotics, thereby shielding bacteria from therapeutic agents [71]. Topical antibiotics remain a mainstay for controlling bacterial burden in DFUs [72]. However, the escalating prevalence of multidrug-resistant pathogens, coupled with stagnant antibiotic development pipelines, has severely limited treatment efficacy [73]. While increasing drug concentrations may transiently enhance antimicrobial activity, such an approach risks dose-dependent toxicity and collateral tissue damage [74]. The resilience of biofilm-embedded bacteria further diminishes drug penetration, creating a therapeutic impasse that sustains chronic infection and delays healing [71].

Generally, diabetics are able to regulate their blood glucose levels via insulin injection [75]. Nevertheless, extended insulin injections may induce side effects like edema, hypoglycemia, and obesity. Besides the aforementioned topical antibiotics and glycemic control, the current clinical approaches for the treatment of diabetic wounds encompass surgical debridement, the application of dressings, and hyperbaric oxygen (O_2_) therapy [72]. However, traditional treatment methods have inevitable disadvantages, including high cost, time consumption, and patient discomfort. Additionally, managing hyperglycemia or performing surgical debridement can have negative effects, such as inducing hypoglycemia or damaging healthy tissue [74]. Consequently, it is of utmost importance to develop novel and effective strategies for promoting the healing of diabetic wounds.

## Overview of nanozymes

In a healthy body, ROS function as critical signaling molecules, while various antioxidant enzymes like SOD, GSH-Px, CAT, and peroxiredoxins maintain ROS at controlled levels to prevent damage to normal cells [76, 77]. Numerous studies have demonstrated that optimal levels of ROS, such as hydroxyl radical (•OH), superoxide anion (O_2_^•−^), and singlet oxygen, play beneficial roles in normal wound healing by promoting cell migration and angiogenesis [78]. In contrast, chronic wounds produce excessive ROS, shifting the redox equilibrium toward sustained oxidative stress. This directly impairs reparative mechanisms and perpetuates tissue damage.

In recent years, nanozymes have been developed for the treatment of diseases, mainly because of their capacity to regulate redox homeostasis and alleviate ROS-dependent inflammation [79]. Various therapeutic approaches using nanozymes have been explored, including antioxidant nanozyme therapies [80], pro-oxidant nanozyme therapies, and combined antioxidant and pro-oxidant nanozyme strategies [81]. In addition to oxidoreductase-like nanozymes, nitric oxide synthase (NOS) mimics show promise in promoting wound healing. They regulate cytokines and recruit immune cells (e.g., monocytes and neutrophils) [72]. This process contributes to biofilm breakdown and reduces bacterial adhesion, leading to biofilm disruption [82].

The most representative nanozymes are classified into several subtypes based on their reactions and the natural enzymes they emulate. These distinct subtypes possess unique active sites that endow them with activities similar to natural enzymes, such as POD, OXD, CAT, and SOD. Given the diverse mechanisms regulating ROS, designing multifunctional nanozymes requires consideration of both enzymatic activity factors and cascade reaction strategies to ensure rational and effective outcomes. By capitalizing on the catalytic activities of multifunctional nanozymes, researchers are able to design cascade reactions customized to tackle specific challenges and attain the desired outcomes in diverse biomedical applications [83].

**Fig. 3:**
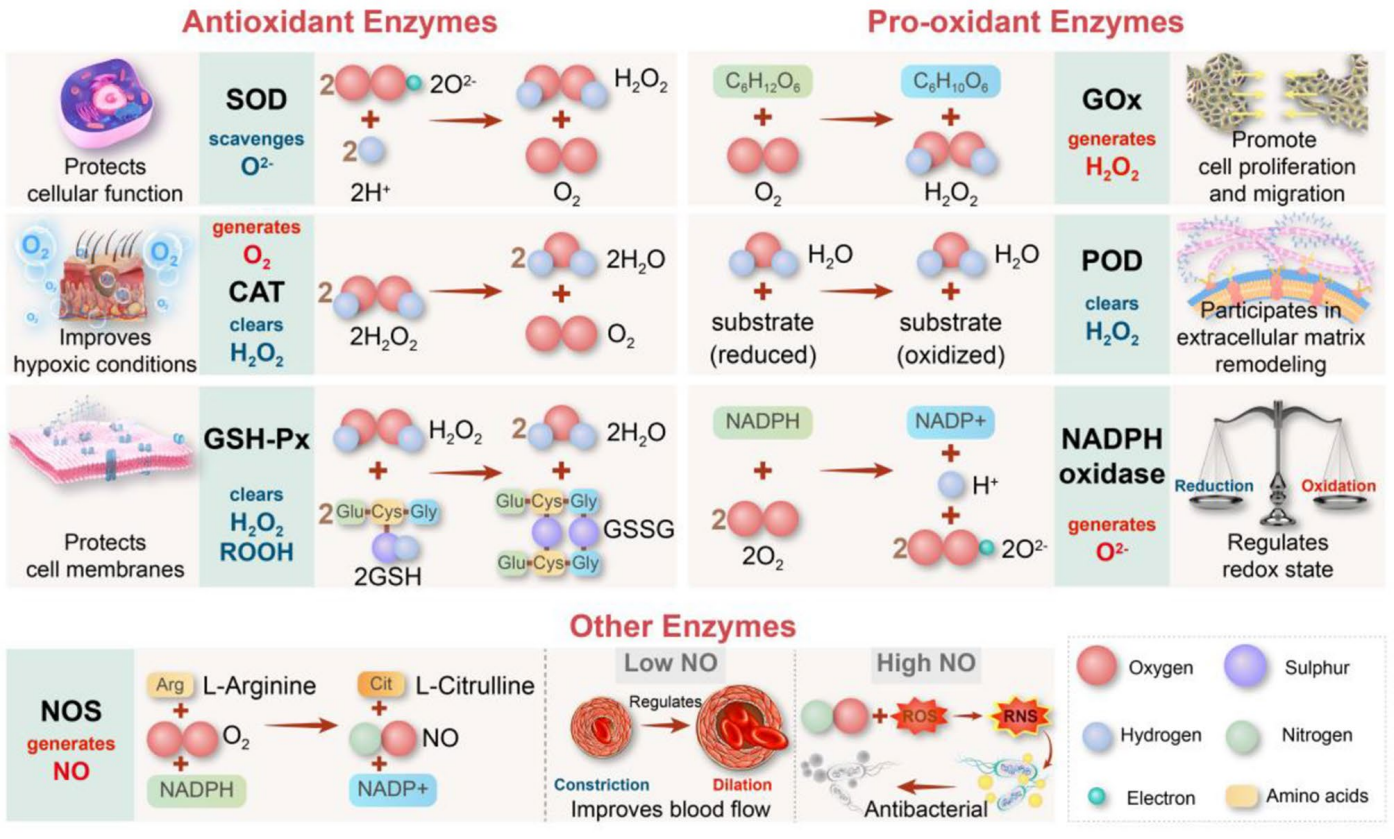
Classification and function of oxidative stress-related enzymes in chronic wound healing. The schematic diagram categorizes these enzymes into three groups: antioxidant enzymes, such as SOD, CAT, and GSH-Px, which neutralize ROS to protect cells; pro-oxidant enzymes, including GOx, POD, and NADPH oxidase, which generate ROS to promote cell proliferation and migration; and other enzymes, like NOS, which regulate blood flow and immune responses

ROS functions as a double-edged sword in numerous diseases. In normal cells, the production and elimination of these reactive species are maintained in a dynamic balance [84]. However, due to their toxic nature, elevated levels of ROS can be leveraged therapeutically to kill pathogens or cancer cells through increased ROS using pro-oxidant approaches [85]. Thus, therapeutic strategy selection, whether to suppress ROS via antioxidant interventions or increase ROS through pro-oxidant approaches, requires context-specific optimization. Critical to this process is the precise spatiotemporal modulation of oxidative stress, thereby governing inflammatory mediator dynamics and microenvironmental homeostasis [86]. In addition, therapeutic nanozyme design must systematically integrate their dual redox functionalities (antioxidant/pro-oxidant) with tissue-specific biocompatibility profiles to achieve targeted therapeutic outcomes. To further explore the versatility and applications of nanozymes, the following sections will discuss and categorize two main types: antioxidant enzymes, including SOD, CAT, and GSH-Px, and pro-oxidant enzymes such as OXD and POD, offering detailed insights into their specific functions and therapeutic potential **(**Fig. 3**)** [87].

### Antioxidant enzymes

Antioxidant enzymes play a vital role in cellular defense mechanisms by alleviating oxidative stress and neutralizing ROS and free radicals inside cells. These enzymes serve to maintain redox homeostasis and safeguard cellular components from oxidative damage [88]. Prominent examples of antioxidant enzymes include SOD, which catalyzes the dismutation of superoxide radicals into O_2_ and H_2_O_2_; CAT, which facilitates the decomposition of H_2_O_2_ into water and O_2_; and GSH-Px, which converts lipid hydroperoxides into their corresponding alcohols and reduces free H_2_O_2_ to water. Based on catalytic activity, antioxidant nanozymes are used to scavenge ROS and alleviate oxidative stress, making them suitable for chronic wounds and inflammatory diseases. However, their insufficient specificity for different ROS types may interfere with normal physiological signaling.

#### SOD

SOD plays a pivotal role as an antioxidant enzyme in the human body. Its primary function is to catalyze the dismutation of O_2_^•−^ into O_2_ and H_2_O_2_, effectively transforming one type of ROS into a more readily decomposable non-radical form. The SOD-like activity effectively removes excess ROS [89]. This process is crucial in alleviating oxidative stress generated from cellular metabolism. Consequently, SOD demonstrates the potential to convert O_2_^•−^ into H_2_O_2_ on a substantial scale, thereby playing a critical role in maintaining cellular redox homeostasis.

**Fig. 4:**
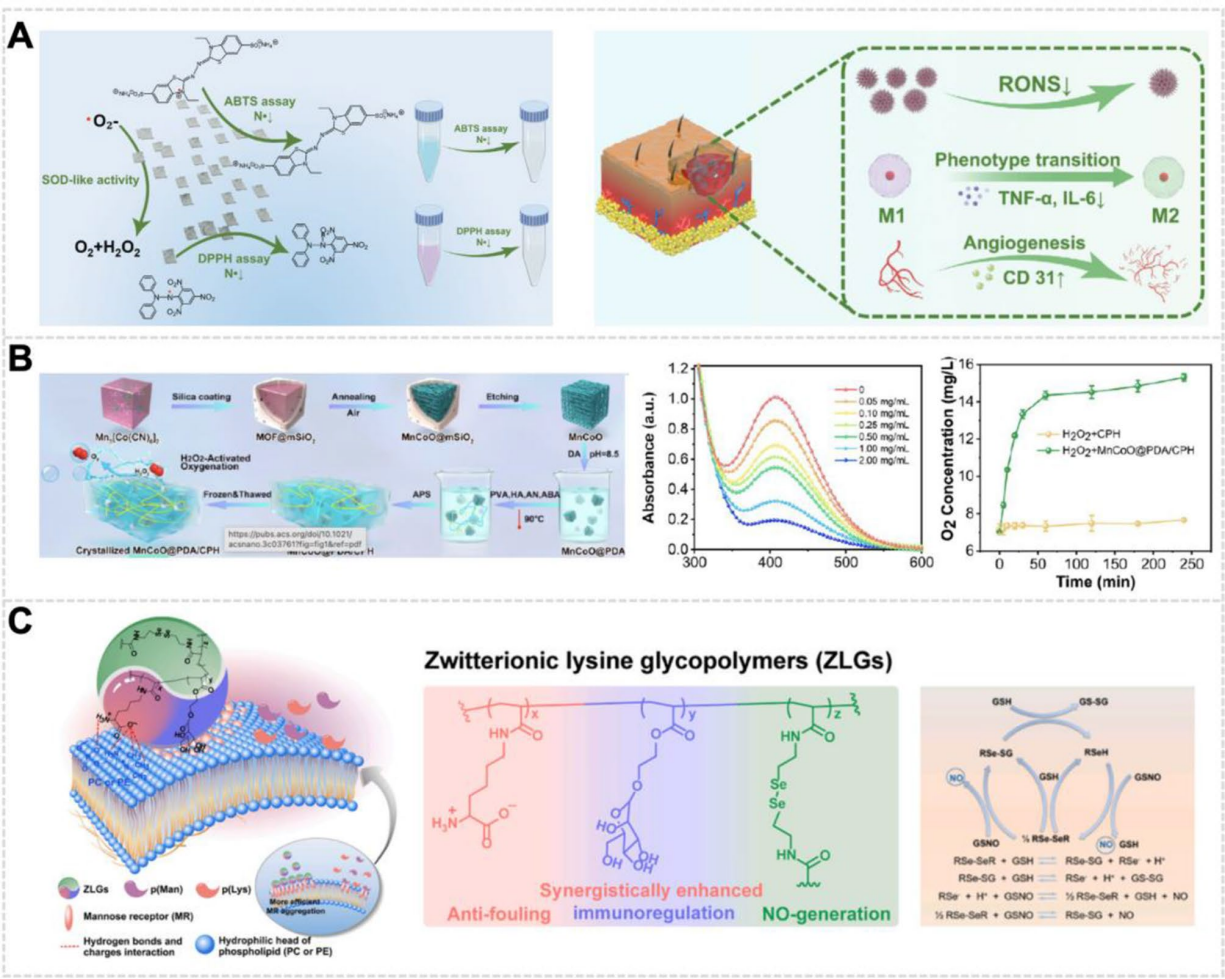
Antioxidant Enzymes and Their Applications. **(A)** Cu_2_Se NSs demonstrate the capability to scavenge ROS and RNS, enhancing macrophage transition and promoting wound healing [92], Copyright 2024, John Wiley and Sons Ltd. **(B)** MnCoO@PDA/CPH hydrogel with H_2_O_2_-activated oxygenation capability, characterized by O_2_ generation and H_2_O_2_ degradation [95], Copyright 2023, American Chemical Society. **(C)** Zwitterionic Lysine Glycopolymers (ZLGs) showcasing interactions with mannose receptors and potential immunoregulatory effects [102], Copyright 2024, Elsevier

SOD nanozymes have demonstrated the capacity to reduce cellular oxidative stress levels [90]. The SOD-like activity of copper-based nanozymes is crucial for their effectiveness in wound repair. This efficacy is further enhanced by their exceptional catalytic performance and near-infrared (NIR)-responsive photothermal properties [91]. He et al. incorporated Cu_2_Se nanosheets, which exhibit SOD-like activity, into F127 hydrogels (Fig. 4A). This integration resulted in a temperature-sensitive nanozyme hydrogel (Cu_2_Se/F127) with enhanced wound healing potential. The Cu_2_Se nanosheets, featuring numerous catalytically active centers, suppress relevant inflammatory pathways, thereby reducing wound inflammatory responses and promoting wound metabolism. Notably, the incorporation of Cu_2_Se nanosheets into the F127 hydrogel does not significantly alter the hydrogel’s intrinsic properties. This hybrid hydrogel exhibits dual benefits: it conforms to the irregular and complex morphology of acute wounds while simultaneously extending the duration of nanozyme activity at the wound site, thereby enhancing the wound-healing process [92].

#### CAT

CAT is a ubiquitous antioxidant enzyme in aerobic organisms. It protects cells from oxidative stress by decomposing H_2_O_2_ into water and O_2_. Primarily localized in peroxisomes, CAT mitigates the harmful effects of excessive ROS accumulation through this critical detoxification mechanism. Hypoxia was initially recognized as a key physiological stimulus for wound repair. However, when it persists beyond optimal levels, it can create a pro-inflammatory environment that hinders the healing process [93]. O_2_ generated by CAT-like activity may alleviate hypoxia. Thus, CAT-like catalysts decompose H_2_O_2_ into O_2_ at the infected site, which is beneficial for treatment.

Nanozymes with CAT-like activity can relieve the hypoxic tumor microenvironment by producing O_2_, which can also be converted into ROS to fight against tumors [94]. Beyond its applications in anticancer treatments, CAT-like activity has also demonstrated potential in anti-inflammatory interventions. Zhao et al. present a biomimetic hydrogel system incorporating CAT-mimicking nanozymes (MnCoO@PDA/CPH) for DFU treatment (Fig. 4B) [95]. This hydrogel system integrates CAT-mimicking nanozymes into a supramolecular network of biocompatible and conductive polymers via polydopamine-mediated assembly. The nanozymes replicate natural catalase activity by decomposing H_2_O_2_ into O_2_, while demonstrating enhanced stability, catalytic efficiency, and cost-effectiveness compared to biological enzymes. Sustained catalytic cycling enables continuous H_2_O_2_ scavenging and O_2_ generation, synergistically mitigating oxidative stress while improving the viability of keratinocytes, fibroblasts, and endothelial cells in hypoxic microenvironments.

#### GSH-Px

GSH-Px belongs to a family of selenoproteins, with selenium at its active site, and is ubiquitously distributed in diverse tissues across the body. The primary function of GSH-Px is to catalyze the reduction of H_2_O_2_ and organic hydroperoxides into water and the corresponding alcohols, respectively [96]. This reaction is essential for protecting cellular components, such as lipids, proteins, and nucleic acids, from oxidative damage. Furthermore, the GSH-Px-like activity can help manage the levels of GSH in the wound microenvironment, potentially enhancing the therapeutic effects of ROS by balancing oxidative and reductive processes [97]. In cancer therapy, the depletion of GSH can increase oxidative stress within tumor cells, leading to apoptosis; this is a mechanism that mirrors the catalytic activity of GSH-Px in promoting cell death [98].

Selenocystamine exhibits GSH-Px-like activities that enable it to catalytically decompose S-nitrosothiols (RSNO) in wound environments, thus generating nitric oxide (NO) [99]. This role as an NO generator not only helps restore NO biogenesis in wounds but also ensures a sustained NO supply [100], crucial for countering NO’s short half-life of mere seconds and maintaining the required working concentration (10^6^-10^9^ M) [101]. The skin’s naturally RSNO-rich microenvironment (~ 35 µM) aids in this restoration, serving as an ideal reservoir for NO biogenesis. Wang et al. utilized selenocystamine, mannose, and lysine as primary building blocks to engineer zwitterionic lysine glycopolymers (ZLGs) through a sequential reversible addition-fragmentation chain transfer polymerization process (Fig. 4C). These ZLGs exhibit GSH-Px-like activity along with antibacterial and macrophage polarization regulatory properties, aimed at enhancing the healing process of bacteria-infected wounds. In the ZLG structure, components are rationally integrated to maintain sustained NO biogenesis. Mechanistically, the NO produced via the decomposition of S-nitrosoglutathione in the presence of ZLGs containing diselenide is mediated by GSH. GSH reduces diselenide bonds (R-Se-Se-R) to generate the primary active species (RSeH/RSe^−^), which promotes the GSH-Px-like decomposition of S-nitrosoglutathione. This endows ZLGs with a potent immunoregulatory capacity, promoting the prompt transition of wound sites through the inflammatory phase following bacterial clearance [102].

### Pro-oxidant enzymes

Pro-oxidant enzymes constitute a functional class of enzymes that generate or amplify intracellular levels of ROS and free radicals. However, their overactivation can lead to oxidative stress and associated pathological conditions [103]. Notable examples of pro-oxidant enzymes include specific OXDs, such as NADPH OXDs, which catalyze the production of superoxide from molecular O_2_ and NADPH; xanthine oxidase, which generates both superoxide and H_2_O_2_ during purine catabolism; and glucose oxidase (GOx), which produces H_2_O_2_ as a byproduct of glucose oxidation [104]. Additionally, certain PODs can exhibit pro-oxidant activity under specific physiological conditions. It is important to note that not all OXDs and PODs function as pro-oxidant enzymes, underscoring the complex nature of redox regulation in biological systems. Classified by catalytic activity, pro-oxidant nanozymes generate ROS to kill pathogens or tumor cells. However, high ROS concentrations can potentially damage healthy tissues, necessitating precise control over their catalytic activity.

#### OXD

OXDs play a pivotal role in biological oxidation processes. These enzymes catalyze oxidation-reduction reactions where molecular O_2_ is the terminal electron acceptor. The primary function of OXDs is to facilitate the transfer of electrons from various substrates to O_2_, typically resulting in the formation of H_2_O_2_ or water as by-products. They are integral to numerous metabolic pathways, including cellular respiration, detoxification mechanisms, and biosynthesis of various compounds [105]. OXDs generally require specific cofactors, such as flavin adenine dinucleotide or metal ions, to sustain their catalytic activity. These enzymes are categorized based on their substrate specificity and the chemical nature of their reaction products. For instance, representative members such as GOx and cytochrome c oxidase exemplify this classification, serving divergent metabolic functions: GOx mediates the oxidation of β-D-glucose into gluconic acid and hydrogen peroxide, a process essential for glucose homeostasis and antimicrobial defense [106], whereas cytochrome c oxidase catalyzes the reduction of oxygen to water and couples this reaction to ATP synthesis by establishing a proton gradient [107]. Recent studies have highlighted the potential of OXD-like nanozymes in antibacterial therapy, as they can directly convert surrounding O_2_ molecules at infection sites into antimicrobial superoxide (O_2_^•−^) and hydroxyl (•OH) radicals [108]. However, native OXD-like nanozymes often demonstrate limited antibacterial efficacy due to insufficient catalytic activity [109]. Consequently, improving the therapeutic performance of these nanozymes has become a key research priority in this field [109]. Notably, O_2_^•−^, a predominant reactive oxygen species (ROS) in tumor therapy, can also be generated by OXD-like nanozymes via oxygen reduction, thus underscoring their dual therapeutic relevance in both antibacterial and anticancer applications.

##### GOx

GOx is a natural enzyme, and its application in diabetic wound healing has gained significant attention due to its unique ability to regulate glucose levels and produce antimicrobial byproducts. During the metabolism of excess glucose in infected diabetic wounds, GOx catalyzes the conversion of glucose into gluconic acid and H_2_O_2_ [106]. This dual action allows for the effective modulation of in situ glucose reduction and ameliorates the hyperglycemic microenvironment of the wound [110]. Simultaneously, it exerts antibacterial properties through the production of H_2_O_2_, which can disrupt bacterial cell integrity and enhance the overall healing process. This process modulates the local pH to a slightly acidic level, which is in accordance with the normal wound pH and promotes healing. However, the rapid removal of glucose by GOx leads to the accumulation of H_2_O_2_. This buildup can generate excess ROS (e.g., O_2_^•−^, •OH), worsening oxidative stress and inflammation in the wound [111].

To address the inherent trade-off between therapeutic benefits and side effects of GOx, researchers have designed glucose-assisted cascade catalytic systems that integrate natural GOx with nanozymes and nanozyme platforms mimicking GOx activity [112]. These systems catalyze the conversion of glucose into gluconic acid and H_2_O_2_ while utilizing POD-like activity to enhance •OH radical generation. This dual action not only improves antibacterial efficacy but also reduces oxidative stress risks. Moreover, these systems enable localized replenishment of H_2_O_2_ at infection sites, circumventing collateral tissue damage induced by systemic administration of exogenous H_2_O_2_ [113]. Simultaneously, gluconic acid produced during the GOx-driven catalytic reaction cascade acidifies the microenvironment, which not only activates the POD-like activity of nanozymes but also synergistically amplifies ROS generation for targeted antibacterial action.

**Fig. 5:**
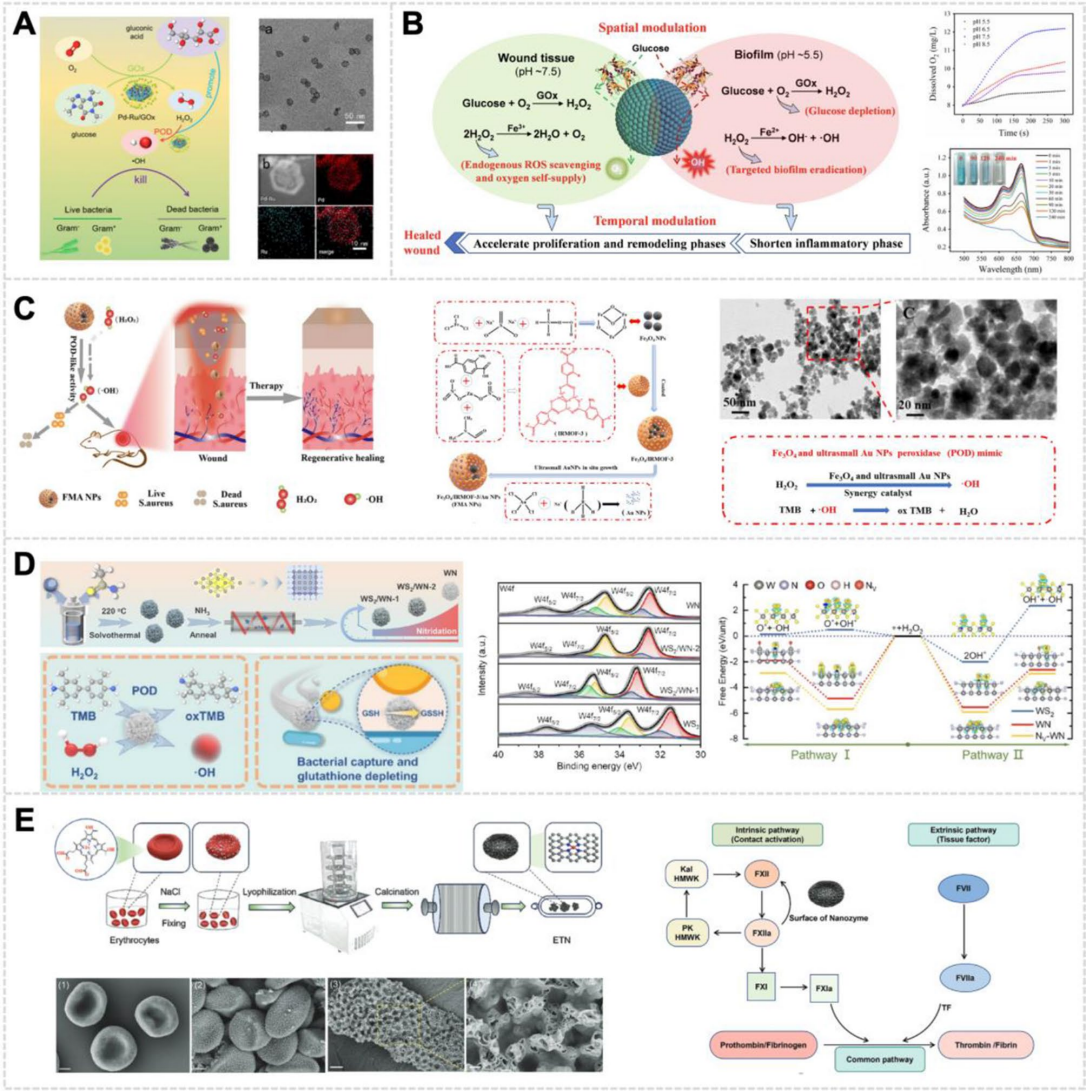
Cascade Catalytic Systems for Diabetic Wound Treatment. **(A)** Pd–Ru/GOx Cascade Nanoreactor demonstrating antibacterial capabilities through glucose-induced ROS generation [114], Copyright 2022, Royal Society of Chemistry. **(B)** Fe_3_O_4_-GOx strategy for wound environment modulation, enhancing O_2_ production and ROS scavenging [112], Copyright 2022, BioMed Central. **(C)** FMA Nanozymes with high POD-like activities for antibacterial wound healing [124], Copyright 2023, BioMed Central. **(D)** Flower-like Artificial POD showcasing nanozyme catalytic mechanisms in antibacterial therapy [133], Copyright 2024, American Chemical Society. **(E)** ETN NPs illustrating procoagulant mechanisms and coagulation pathway activation [135], Copyright 2024, John Wiley and Sons Ltd

The development of innovative nanozyme-based therapies has improved the efficacy of diabetic wound healing treatments. These approaches combine multiple enzymatic activities and enhance glucose sensing capabilities. Zhu et al. developed a Pd-Ru/GOx cascade reactor using two-dimensional (2D) Pd-Ru nanosheets with glutaraldehyde coupling chemistry covalently grafted to GOx, which converts glucose into •OH capable of killing bacteria (Fig. 5A) [114]. When SOD, CAT, and GOx are all highly active, the system can generate O_2_ by catalyzing the H_2_O_2_ produced during glucose oxidation. This process regulates the O_2_ balance within the wound and mitigates the toxicity of GOx, thereby enabling the synergistic repair of diabetic wounds [39]. Du et al. synthesized an Fe_3_O_4_ nanozyme with POD-like and GOx activities through electrostatic coating and covalent modification of GOx, which could effectively eliminate biofilm infection in diabetic wounds and accelerate healing (Fig. 5B) [112].

#### POD

POD is a natural enzyme found in various organisms, including plants, humans, and bacteria [78]. It belongs to a large enzyme class that catalyzes substrate oxidation in the presence of peroxides, primarily H_2_O_2_ and certain organic hydroperoxides [115]. This enzyme family exhibits broad-spectrum antibacterial activity, high catalytic efficiency, and remarkably low bacterial resistance development [109]. Building on the catalytic versatility of POD, chemodynamic therapy (CDT) has emerged as a promising strategy for disease treatment by producing highly toxic ROS through Fenton-like reactions [116]. In this context, POD-like enzymes play a crucial role by catalyzing H_2_O_2_ into highly cytotoxic •OH, thereby increasing oxidative stress in bacteria [117]. While H_2_O_2_ is a common antibacterial agent effective in destroying bacterial proteins and DNA [118], its efficacy is concentration-dependent. High concentrations required for bactericidal effects can potentially damage healthy tissues, exacerbate inflammation, and even harm the central nervous system [119]. To address this challenge, POD-like nanomaterials have been developed to generate toxic •OH under low levels of H_2_O_2_ [120]. Therefore, enhancing the POD-like activity of nanomaterials is an effective antibacterial strategy.

The widespread use of iron in POD-like nanozyme research is attributed to its biological similarity to natural PODs, exceptional catalytic activity in Fenton-like reactions, and multifunctional versatility in enzymatic activities. Notably, many natural PODs, such as horseradish peroxidase (HRP), contain iron in their active sites, further underscoring the element’s significance in enzymatic processes. Since the discovery of Fe_3_O_4_ NPs’ POD-like activity, iron-based nanomaterials have become a cornerstone in the field of nanozymes [121], showing great potential in tumor therapy [122] and combating antimicrobial resistance [123].

Iron’s abundance, biocompatibility, and tunable properties make it an ideal candidate for nanozyme development. For instance, Liu et al. reported an innovative antibiotic-independent hybrid nanozyme for antimicrobial wound healing (Fig. 5C). This system, composed of ultra-small Au NPs grown in situ on MOF-stabilized Fe_3_O_4_ NPs, displayed synergistic POD-like activities. The hybrid nanozymes generated a significant amount of •OH at low doses of H_2_O_2_. The cascade reaction between Au and Fe_3_O_4_ NPs accelerated the reaction rate, improving antibacterial efficiency. This bimetallic nanozyme system achieved excellent therapeutic effects at low H_2_O_2_ concentrations and showed significant potential in infected wound healing [124]. Nevertheless, the catalytic efficiency of iron oxide NPs is frequently lower in comparison with that of natural enzymes due to lower utilization of iron atoms for catalysis. In order to address this limitation, single-atom catalysis has been utilized to generate precise active sites that mimic the iron-porphyrin coordination of natural enzymes, thus significantly enhancing the efficiency of iron and the specific activity of nanozymes [125]. Through diverse synthesis methods and combinations, including single-atom catalysts and bimetallic systems, researchers have continuously optimized iron-based POD nanozymes. These structural optimizations have led to superior performance in antibacterial applications and wound healing. Fine-tuning the coordination environment of iron, especially in Fe-N configurations, enables precise modulation of catalytic behavior. This often leads to greater efficacy than that of natural enzymes.

After exploring iron-based nanozymes, research has progressed to single-atom catalysts and the strategic incorporation of various metal sites. These advancements have led to significant improvements in POD-like performance. Studies on incorporating diverse metal sites (Fe, Co, Mn, Ni, Cu, and Zn) into metalloporphyrin ligands have revealed intriguing enzymatic behaviors, particularly exemplified by PCN-222(Fe), which serves as an outstanding model for POD-like activity [126]. Researchers use carbon-based substrates to support single-atom nanozymes [127]. By varying metal atom species and coordinating ligand types, they can control the geometric and electronic configurations of active sites. This optimization enhances enzyme-mimicking behavior [128]. For example, through careful adjustment of Fe-N coordination dynamics, the synthesized FeN5 structures exhibit rate constants that surpass those of conventional commercial nanozymes [129]. While metal sites are crucial, research has revealed that the overall performance depends on both metallic and nonmetallic active centers, highlighting the complexity of these systems [130], including N vacancies, doping, coordination, and nitride formation [131]. Nitrogenation has emerged as a critical strategy for enhancing nanozyme efficiency in transition metal materials. This is exemplified by the nitridation of TiO_2_ to TiN, which significantly enhances the material’s capabilities, with the degree of nitridation directly correlating with improved POD-like performance [132].

Building upon the understanding of nitrogen’s role in nanozyme architecture, recent research has further explored its impact on specific materials, as demonstrated in the study of tungsten nitride (WN) nanozymes. The investigation by Yang et al. highlights the crucial role of nitrogen in enhancing the POD-like activity of WN (Fig. 5D). Density-functional theory computations reveal that nitride formation in WN confers nanozyme functionality, while precise control of nitrogen vacancies stabilizes the OH* lattice adsorption state. This stabilization accelerates desorption kinetics and lowers the energy barrier for ROS generation, thereby amplifying catalytic efficacy. WN’s efficient catalytic prowess initiates a cascade of free radicals that swiftly targets and sterilizes pathogen surfaces, effectively preventing biofilm formation and halting bacterial proliferation. This research not only elucidates the mechanism behind WN’s enhanced POD-like activity but also showcases its promising application in combating bacterial infections [133].

##### Heme peroxidases

Cytochrome c peroxidase, myeloperoxidase, and HRP belong to the heme peroxidase superfamily. They share heme-containing active sites and can catalyze peroxide-dependent oxidation reactions. Cytochrome c peroxidase is a mitochondrial enzyme essential for the electron transport chain. It participates in oxidation-reduction reactions that support cellular respiration and energy metabolism. Myeloperoxidase, predominantly found in neutrophils and other white blood cells, catalyzes the production of hypochlorous acid from H_2_O_2_ and chloride ions. This process generates a potent antimicrobial agent. HRP, primarily extracted from horseradish roots, catalyzes the oxidation of various organic substrates in the presence of H_2_O_2_. Among these enzymes, HRP has gained particular prominence in practical applications due to its high catalytic activity, stability, and versatility, making it a valuable tool in biochemical research, diagnostic assays, and biotechnological applications.

HRP’s active center consists of a heme group. The heme group is the principal component of hemoglobin, which is abundantly found in erythrocytes. Remarkably, each erythrocyte contains around 260 million hemoglobin molecules [134], making it an optimal source of inherent single-atom iron for nanozyme synthesis that mimics HRP activity [135]. The heme group typically comprises an Fe atom coordinated with four N atoms in a planar arrangement. This planar configuration is a defining characteristic of the heme prosthetic group and plays a critical role in enzymatic reactions, such as the catalytic decomposition of H_2_O_2_. The H_2_O_2_ molecules can associate with the iron atom within the heme group. During the catalytic process, the iron atom facilitates the decomposition of H_2_O_2_ into water and molecular O_2_ through electron transfer. Motivated by natural design, Wang et al. proposed an inventive erythrocyte-templated strategy in which the intracellular hemoglobin-rich content was used as an iron resource for the preparation of iron single-atom nanozymes [135]. The inherent biochemical environment provided by erythrocytes not only facilitates the uniform distribution of iron at the atomic level but also enhances the stability and reactivity of the nanozymes under physiological conditions. For example, erythrocytes were used as uniform templates to synthesize nanozymes with atomically dispersed FeN_4_ active sites derived from hemoglobin. This was achieved through a consecutive process of cell fixation, porous salinization, and high-temperature carbonization, resulting in enhanced POD-like activity. The synthesized nanozymes efficiently catalyze H_2_O_2_ to produce •OH, with their catalytic performance further enhanced under NIR exposure (Fig. 5E) [135].

## Mechanisms of nanozymes in promoting wound healing

Nanozymes demonstrate remarkable capabilities in simultaneously addressing multiple facets of tissue regeneration and wound healing [39]. Diabetic wounds are highly susceptible to oxidative stress due to the overproduction of ROS and insufficient antioxidants [84]. Nanozymes with SOD-like, CAT-like, or GSH-Px-like activities can scavenge excess ROS, thereby reducing oxidative stress and promoting a healing-conducive environment. Antibacterial activity is another crucial aspect. In the diabetic wound microenvironment, characterized by high concentrations of H_2_O_2_, nanozymes with POD-like activity can catalyze the conversion of H_2_O_2_ to •OH. These radicals effectively kill bacteria, eliminating infectious pathogens [113]. Additionally, certain nanozymes address glucose regulation through GOx-like activity, converting glucose in the wound into gluconic acid and H_2_O_2_. This not only reduces blood sugar levels but also produces H_2_O_2_ as a substrate for further reactions, potentially enhancing bacterial removal and alleviating the hyperglycemic environment [136]. Some nanozymes can generate therapeutic gases, further contributing to the healing process through various mechanisms. These nanozymes enhance multiple aspects of tissue regeneration, including promoting dermal reconstruction, stimulating angiogenesis, and facilitating collagen deposition. By simultaneously targeting bacterial infections, oxidative stress, glucose regulation, and tissue reconstruction, nanozymes provide an integrated approach to address the complex nature of diabetic wound healing. This multifaceted strategy offers promising potential for enhancing the healing process in diabetic and other chronic wounds.

### Antibacterial activity

The primary antimicrobial mechanism of nanozymes centers on their ROS-mediated catalytic therapy. This process generates various ROS species, including hypochlorous acid, O_2_^•−^, H_2_O_2_, and •OH, which effectively eliminate bacteria through multiple pathways [137]. ROS exhibit antimicrobial properties by disrupting bacterial cellular components, including cytomembranes, proteins, and DNA, resulting in compromised cellular integrity. ROS-mediated lipid peroxidation of bacterial cell membranes causes increased membrane permeability and subsequent cellular lysis. ROS-induced protein oxidation results in denaturation and functional impairment, particularly affecting essential enzymes and structural proteins. Furthermore, ROS cause DNA damage through oxidative modifications and strand breakage, disrupting normal cellular processes and potentially inducing cell death. The cumulative effect of these oxidative damages overwhelms bacterial antioxidant defense mechanisms, ultimately leading to bacterial inactivation or death [137].

OXD and POD nanozymes effectively prevent wound infection through enhanced ROS generation [136]. Hu et al. synthesized ultra-fine Au NPs in situ on ultra-thin 2D MOF, exhibiting POD-like activity. These nanozymes effectively catalyze the decomposition of H_2_O_2_ into cytotoxic •OH, demonstrating potent antimicrobial activity against *S. aureus* and subsequently promoting wound healing [138]. Furthermore, investigations have revealed that cascade nanozyme systems comprising Au and Pt nanozymes can initiate sequential enzymatic reactions to generate cytotoxic ROS through glucose catalysis [139]. This sophisticated system not only produces ROS but also potentially modifies the local microenvironment, thereby enhancing its antimicrobial efficacy. Additionally, certain nanozymes exhibit photocatalytic properties, utilizing light energy to generate electrons under illumination, which subsequently amplifies ROS production and bacterial elimination. The synergistic integration of these multiple mechanisms—including chemical catalysis, physical interactions, and photocatalytic activity—renders nanozymes particularly effective against a broad spectrum of bacterial pathogens, including antimicrobial-resistant strains. Given their unique properties, nanozymes show great potential as catalysts for CDT-based sterilization. Particularly, they can effectively utilize endogenous glucose in the microenvironment of diabetic chronic wounds. Their multifaceted antibacterial mechanisms, coupled with inherent stability and tunable properties, position nanozymes as a promising therapeutic strategy for addressing bacterial infections in chronic wounds.

Beyond ROS generation, nanozymes demonstrate antimicrobial efficacy through direct physical interactions with bacterial cells. Their high surface area-to-volume ratio and nanoscale dimensions facilitate direct contact with bacterial cell membranes, potentially inducing mechanical damage. Furthermore, electrostatic interactions between charged nanozyme surfaces and bacterial cell components can result in membrane disruption. Certain nanozymes can penetrate bacterial cell membranes, inducing intracellular damage [140, 141].

### Antioxidant activity

In diabetes patients, elevated blood glucose levels frequently result in excessive accumulation of ROS, which can cause cellular damage, inflammation, and delayed wound healing [78]. Oxidative stress and decreased antioxidant capacity cause redox imbalance and exacerbate damage to cells and tissues, a major contributor to the non-healing of diabetic wounds [142]. Redox imbalance is usually associated with an excessive accumulation of oxidative products and decreased activity of antioxidant enzymes. This oxidative stress burden causes irreversible damage to crucial wound healing components, including proteins, lipids, and DNA structures [143].

Nanozymes promote wound healing through their antioxidant properties via multiple mechanisms. Primarily, they exhibit biomimetic properties of natural antioxidant enzymes, such as SOD and CAT, efficiently catalyzing the conversion of deleterious O_2_^•−^ and H_2_O_2_ into benign O_2_ and H_2_O, thereby attenuating oxidative stress and maintaining tissue integrity. In diabetic wounds, where chronic inflammation impedes healing, nanozymes’ ROS-scavenging capability effectively mitigates inflammatory responses [144]. Furthermore, nanozymes reduce oxidative stress and inflammatory mediators, creating an optimal microenvironment for cellular proliferation and migration. This accelerates the regeneration of dermal cells and tissues [88]. Notably, nanozymes facilitate angiogenesis through antioxidant-mediated pathways, enhancing O_2_ perfusion and nutrient delivery to the wound site, thus supporting tissue repair processes [145]. Through these interconnected mechanisms, nanozymes demonstrate therapeutic efficacy not only through direct participation in wound healing via their antioxidant capabilities but also through the orchestration of multiple healing pathways.

Recent advances in nanozyme research have revealed their significant role in modulating redox-sensitive transcription factors and associated signaling pathways. Emerging studies have provided compelling evidence regarding the specific molecular mechanisms underlying these interactions. A pivotal investigation demonstrated that cerium oxide NPs effectively protect chondrocytes and cartilage against damage through activation of the Nrf2/HO-1 signaling pathway, exhibiting potent antioxidant and anti-inflammatory properties [146]. In a separate study, researchers developed taxifolin (Tax)-iron nanozymes (Fe-Tax) by conjugating Tax with iron ions, which demonstrated dual CAT and SOD-like activities in the gastrointestinal environment. The investigation revealed that Fe-Tax nanozymes efficiently scavenged ROS and reactive nitrogen species (RNS), thereby attenuating oxidative damage, inflammation, and cellular apoptosis in vitro. Moreover, in an ethanol-induced gastric ulcer model, Fe-Tax significantly ameliorated tissue inflammation and gastric mucosal damage. This effect was achieved through the modulation of multiple signaling pathways, including Nrf2, NF-κB, Bax/Bcl-2, and vascular endothelial growth factor (VEGF) cascades [147]. These comprehensive studies provide substantial evidence demonstrating the mechanism by which nanozymes exert their anti-inflammatory and cytoprotective effects through the regulation of specific redox-sensitive pathways.

### Gas therapy through gas-generating nanozymes

Diabetic wounds frequently progress to chronic lesions due to their severely dysregulated inflammatory microenvironment, which is characterized by excessive ROS production and localized hypoxia [148]. Anti-inflammatory gases, such as H_2_, H_2_S, and NO, are used to combat inflammatory states and promote the healing of diabetic wounds. These gases typically possess reducing properties that are capable of scavenging ROS, thereby alleviating oxidative stress. Gas-generating nanozymes have emerged as a promising therapeutic strategy for chronic and refractory wounds, particularly those associated with diabetes. Conventional O_2_ therapies, including hyperbaric O_2_ treatment and topical gaseous O_2_ administration, frequently demonstrate limited efficacy due to insufficient tissue penetration [149]. Gas-generating nanozymes address these limitations by facilitating site-specific, controlled release of therapeutic gases, particularly NO and O_2_. NO contributes to multiple therapeutic effects, including vasodilation, angiogenesis promotion, and antimicrobial activity, while O_2_ supplementation supports cellular metabolism, collagen synthesis, and enhanced immune responses. Additionally, NO possesses natural antibacterial capabilities, inhibiting and killing a variety of bacteria, including drug-resistant strains [150]. The superior catalytic efficiency, stability, and specificity of nanozymes enable precise modulation of the wound microenvironment while simultaneously addressing both oxidative stress and hypoxia. This dual-action approach facilitates accelerated wound healing, reduces infection risk, and enhances tissue regeneration. Furthermore, the adaptable properties of nanozymes permit targeted delivery and controlled gas release kinetics, optimizing therapeutic outcomes in chronic wound management.

#### Hypoxia modulation strategies

While acute hypoxia initially functions as a physiological signal promoting wound healing, chronic hypoxic conditions perpetuate pro-inflammatory states and impede the healing process [93]. The therapeutic significance of O_2_ administration in infected wounds derives from its dual capacity to ameliorate hypoxia and modulate macrophage phenotype plasticity [151]. Mechanistically, O_2_ therapy regulates wound healing through modulation of hypoxia-inducible factor 1α (HIF-1α) expression, which orchestrates the phenotypic transition of pro-inflammatory M1 macrophages toward anti-inflammatory M2 phenotypes. The suppression of HIF-1α expression facilitates M2 macrophage recruitment, tissue infiltration, and mobility while promoting the secretion of anti-inflammatory cytokines [152]. Additionally, O_2_ bubbles generated within the wound microenvironment enhance catalyst penetration, thereby potentiating pathogen elimination at the infection site.

**Fig. 6:**
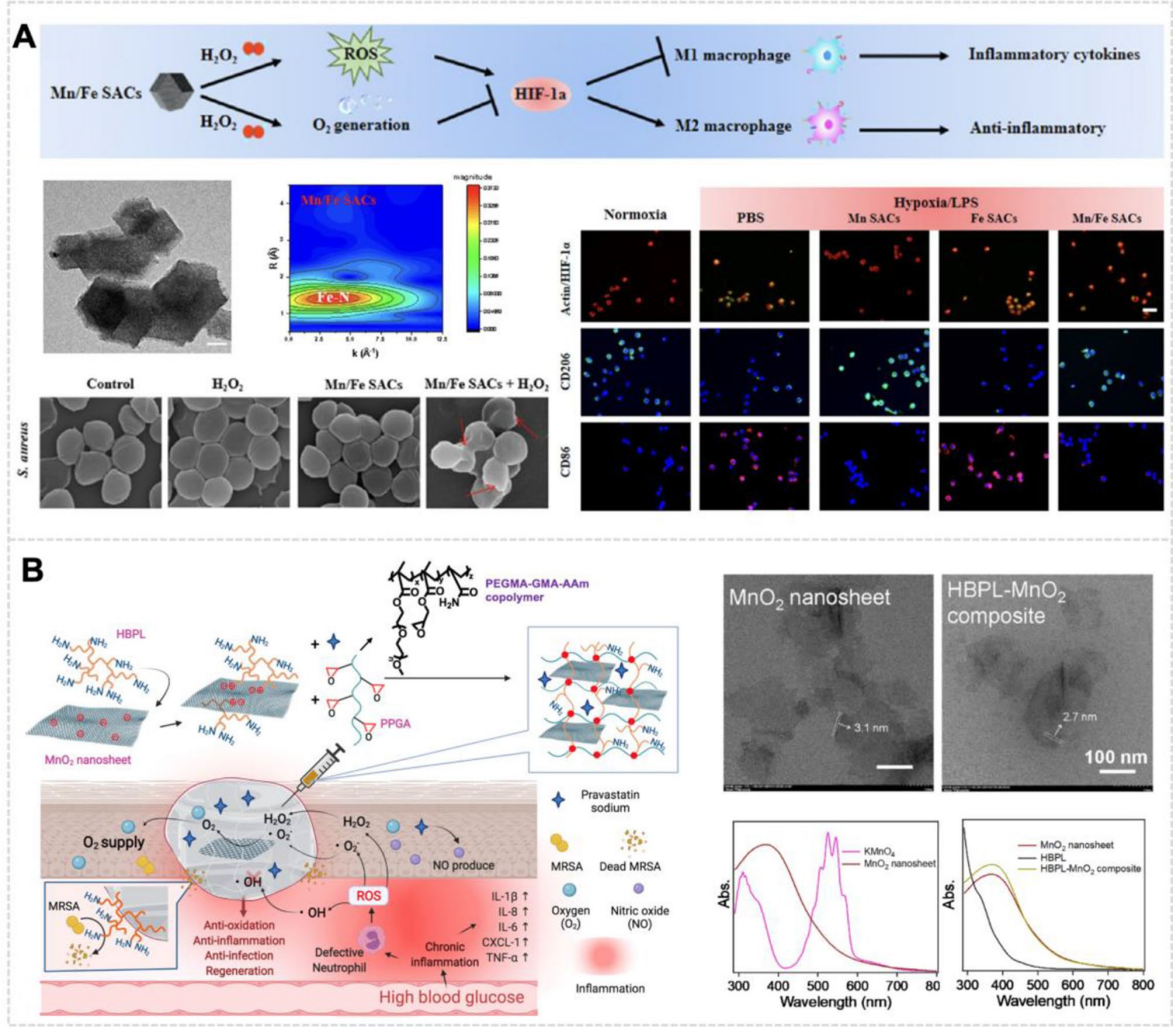
Gas Therapy for Wound Healing. **(A)** Mn/Fe SACs demonstrating O_2_ generation and macrophage polarization regulation through H_2_O_2_ catalysis. Structural characterization and cellular response analysis reveal the catalysts’ potential in modulating inflammatory processes [156], Copyright 2023, American Chemical Society. **(B)** Multifunctional hydrogel with NO-producing and O_2_-generating capabilities, designed for antibacterial treatment and wound healing in diabetic MRSA-infected wounds [148], Copyright 2022, Elsevier

CAT-like nanozymes can catalyze the conversion of H_2_O_2_ to O_2_ at infection sites, providing an effective therapeutic strategy for wound-associated infections. The sustained O_2_ release alleviates hypoxic conditions within bacterial biofilms, maintaining optimal O_2_ homeostasis that supports initial wound repair while preventing the detrimental effects of chronic hypoxia [153]. Various inorganic nanozymes, including calcium peroxide [154] and manganese dioxide (MnO_2_) nanosheets [155], have demonstrated the capability to convert excessive ROS to O_2_ in diabetic wounds. In a pioneering study by Zhang et al., novel manganese-iron dual single-atom catalysts (Mn/Fe SACs) were synthesized through a hydrothermal/pyrolysis approach (Fig. 6A). Manganese functions as a catalyst, promoting H_2_O_2_ decomposition into water and O_2_, mimicking the natural CAT enzyme, hence exhibiting CAT-like activity. Within the Mn/Fe SACs structure, the Fe-N_4_ moiety demonstrated POD-like activity, while the Mn-N_4_ configuration primarily exhibited CAT-like activity. Significantly, Mn/Fe SACs possessed GSH-depleting capabilities, enhancing CDT efficacy and promoting infected wound healing. In comparison to conventional antimicrobial agents, the Mn/Fe SACs-mediated H_2_O_2_ catalytic therapy demonstrated dual advantages: effective pathogen elimination and resistance prevention. Moreover, Mn/Fe SACs exhibited glutathione OXD-mimicking activity, further augmenting their anti-infection efficacy during the wound healing process [156].

#### NO-Mediated regeneration

The accumulation of bacterial debris at infection sites, characterized by substantial release of lipopolysaccharides and peptidoglycans, triggers immune cell activation and significantly intensifies inflammatory responses [157]. In chronic wounds, persistent inflammation impairs NO biogenesis, compromising both pathogen elimination and vascular homeostasis, particularly affecting angiogenesis and vascular remodeling processes [158]. NO, functioning as a pleiotropic intracellular messenger, serves as a critical mediator in inflammatory regulation. Mechanistically, NO modulates macrophage phenotype transition from pro-inflammatory to anti-inflammatory states through the regulation of intracellular mitochondrial metabolism [159]. NO exhibits an excellent antimicrobial effect with unique properties [160]. Firstly, NO reacts with ROS to produce RNS [161]. The oxidative and nitrosative/nitrative reactions of RNS cause DNA deamination, bacterial protein damage, and lipid peroxidation in microorganisms. Such damage disrupts the cellular structure and function, leading to bacterial death [162]. Secondly, it has been reported that NO is capable of inhibiting biofilm formation and clearing mature biofilms [163]. Thirdly, elevated NO concentrations facilitate vascular normalization, thereby restoring physiological perfusion and tissue oxygenation [164]. In the wound-healing stage, NO plays a crucial role in inflammation, cell proliferation, migration, and angiogenesis [165]. Based on these mechanistic insights, Tu et al. developed a multifunctional hydrogel system through the cross-linking of poly(PEGMA-co-GMA-co-AAm) nanozymes with HBPL-MnO_2_ nanosheets (Fig. 6B). This advanced platform integrates multiple therapeutic functions, including antibacterial activity, ROS generation, and NO production capabilities [148].

### Glucose regulation

The regulation and management of the hyperglycemic microenvironment in diabetic wounds may provide novel therapeutic strategies for chronic diabetic wound healing. Among various glucose regulatory mechanisms, GOx-mediated catalysis represents a classical approach for in situ glucose concentration reduction [166], particularly suited for modulating the hyperglycemic wound microenvironment. Mechanistically, GOx catalyzes the conversion of glucose to glucuronic acid with concurrent H_2_O_2_ production [167]. Consequently, effective management of GOx-generated byproducts is crucial for overcoming the limitations associated with GOx-based therapeutics. To address these challenges, Yu et al. engineered a glucose/ROS cascade-responsive nanozyme system for diabetic wound treatment. This advanced platform, constructed through Ce-driven coassembly with a dual-ligand system (comprising alendronic acid and 2-methylimidazole) and GOx, exhibits both SOD and CAT mimetic activities, effectively scavenging excess ROS. Significantly, this nanozyme system catalyzes the conversion of excess H_2_O_2_ generated during glucose oxidation to produce O_2_, thereby mitigating GOx-associated cytotoxicity and achieving synergistic wound repair in diabetic conditions [81].

## Classification of stimuli-responsive nanozymes

### Physical stimuli-responsive nanozymes

#### NIR light-responsive nanozymes

Light, as a tunable stimulus, possesses the advantages of convenient manipulation and requiring no pretreatment. NIR, encompassing wavelengths from approximately 750 to 2500 nm, offers superior tissue penetration and minimal biological damage compared to visible light. These photonic advantages have led to the development of optically responsive intelligent platforms. These platforms use light-matter interactions to enable on-demand therapeutic functions. Jia et al. established a photothermal-chemotherapy synergy system. It uses 808 nm NIR irradiation for controlled doxorubicin release, enabling targeted tumor therapy [168]. Building on advances in light-controlled drug delivery, Yang et al. engineered photoactivated dual-network hydrogels. These hydrogels combine dynamic covalent bonds with non-covalent interactions. As a result, they achieve self-healing, shape adaptability, and mechanical robustness [169]. Further expanding the biomedical applications, Chen et al. developed a rapid self-gelling hydrogel with biphasic thermal dynamics under laser stimulation. This hydrogel shifts from gradual heating to rapid temperature rise, allowing for simultaneous wound regeneration and scar suppression in one treatment [170]. NIR light-responsive nanozymes are a new type of artificial nanomaterial. They combine enzyme-like catalytic activity with responsiveness to NIR light. These nanozymes can adjust their activity and initiate specific reactions when exposed to NIR light, allowing precise control over enzymatic processes. Their controllable activation mechanism, combined with the deep tissue penetration capability of NIR light, makes them particularly promising for biomedical applications such as targeted cancer therapy, antimicrobial treatment, and wound healing.

**Fig. 7:**
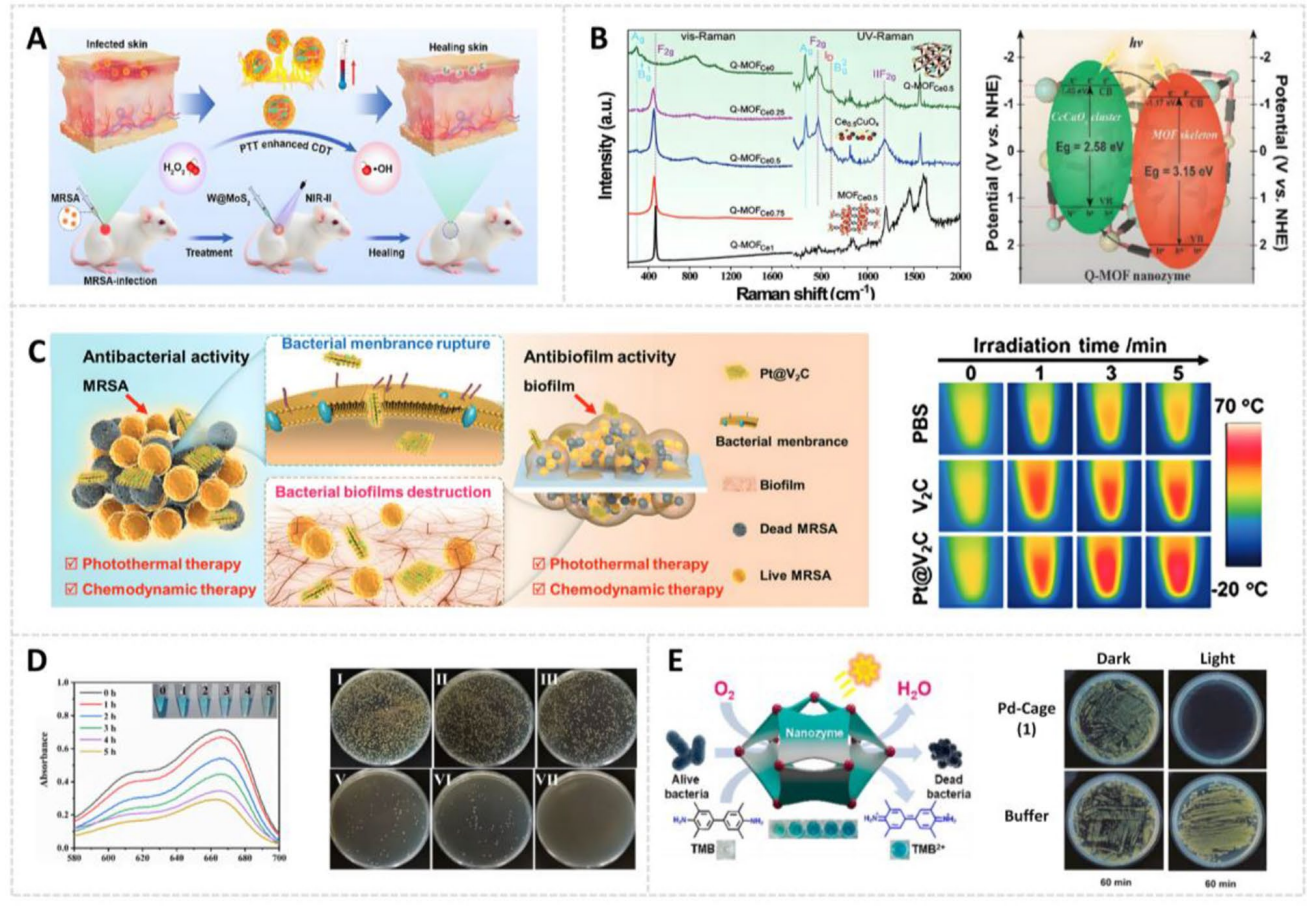
Photothermal response nanozyme for wound healing. **(A)** Schematic demonstrating how 1064 nm laser irradiation enhances the POD-like activity of MoWS_2_, promoting healing of *S. aureus*-infected abscesses in vivo [175], Copyright 2024, Elsevier. **(B)** Normalized POD-like kinetic parameters for various nanozymes and a schematic illustrating the photoactivity mechanisms of Q-MOF nanozymes [179], Copyright 2022, John Wiley and Sons Ltd. **(C)** Illustration showing the mechanisms of action for Pt@V_2_C in disrupting bacterial membranes and biofilms through photothermal and chemodynamic therapies [183], Copyright 2024, John Wiley and Sons Ltd. **(D)** CuS@Pt-Au/Apt NPs combine photothermal therapy and chemodynamic activity, demonstrating antimicrobial efficacy through distinct inhibition zones on 9-cm LB agar plates with infected wound samples across treatment groups [190], Copyright 2023, Elsevier. **(E)** Schematic of the overall catalytic process, highlighting the conversion of glucose to reactive species that target bacteria, enhancing antibacterial efficacy [192], Copyright 2020, American Chemical Society

Deep tissue bacterial infections, particularly MRSA, complicate clinical therapy due to ineffective debridement and frequent recurrences [171]. MoS_2_, a well-known photothermal agent with demonstrated biocompatibility, has been utilized in antibacterial applications. Its efficacy within the NIR region I (NIR-I, 650–900 nm) window for anti-MRSA therapy is regarded as highly effective for photothermal ablation [172]. However, most photothermal therapy (PTT) systems developed with 1T-MoS_2_ require in vivo sterilization temperatures exceeding 55 °C, risking damage to normal skin tissues [173]. Furthermore, MoS_2_’s limited NIR-I tissue penetration and single treatment mode result in inadequate therapeutic effects on deep-tissue infections [174]. To overcome these limitations, Du et al. developed a novel antibacterial strategy combining photothermal and enzyme catalysis. They prepared a defect-type hybrid 2H-MoS_2_ nanozyme (MoWS_2_) using a hydrothermal method. Tungsten ion doping of 2H-MoS_2_ endowed MoWS_2_ with an enhanced photothermal effect (36.9%) in the NIR-II window and enhanced POD-like activity (Fig. 7A). This approach allows for a more effective, milder, and deeper antibacterial strategy. The researchers controlled the maximum temperature to approximately 50 °C to increase the POD-like activity without harming normal skin. The combination of PTT and CDT exhibited a more potent bactericidal effect while preventing high-temperature damage. In vitro experiments showed that MoWS_2_ achieved efficient bactericidal activity and biofilm clearance through hyperthermia and ROS generation. Deep MRSA infection experiments revealed that MoWS_2_ rapidly removed bacteria from subcutaneous infected tissues through PTT and CDT, facilitating abscess resolution and accelerating infected wound healing [175].

MOFs serve as a versatile platform for the biomimetic design of nanoenzymes. They offer controllable pore structures, diverse metal centers, and customizable organic ligands [176]. These MOF-based nanozymes can be activated by external stimuli to enhance their activity at disease sites, enabling targeted disinfection of bacteria [177]. Huang et al. designed MOF and managed the decarboxylation of linkers to create MOF-derived POD nanozymes to combat pathogenic bacteria (Fig. 7B). Cooperative derivatization formed heterojunction-like quasi-MOF interfaces with multivalent active sites and improved visible-light photochemical characteristics [178]. Significantly, the improved photoactive POD mimetic activity of Q-MOF-Ce_0.5_ nanosheets was mainly achieved by carefully creating oxygen vacancies, using multiple redox cycles, and optimizing the photosensitive energy band structure at the large heterojunction-like interface. This structural design dramatically enhanced the efficacy of photo-guided on-demand antibacterial treatments both in vitro and in vivo. The integrated structural and functional features enabled the POD-like Q-MOF-Ce_0.5_ to generate sustained ROS under visible light irradiation, effectively eradicating surface-adhered bacteria [179].

In view of the challenge of multidrug resistance to antibiotics, non-antibiotic-dependent antibacterial strategies hold promise for anti-infective therapy [180]. PTT has emerged as a promising therapeutic modality due to its remarkable thermal effects, precise targeting, and minimal adverse effects [181]. While many photothermal agents show potential, current materials require enhancements in therapeutic efficacy, tissue penetration ability, photothermal conversion efficiency, and laser compatibility. V_2_C MXene-based nanomaterials have exhibited remarkable biocompatibility and photothermal conversion efficiency in PTT applications (Fig. 7C). However, V_2_C MXene’s limited absorption spectrum in the NIR-I window impedes its ability to reach deep tissues and achieve complete bacterial eradication with single-agent treatments. Pt NPs, as precious metal-based inorganic nanozymes, have demonstrated the capability to produce highly toxic •OH through multi-enzyme-like activities (OXD-like and POD-like activity) via CDT, enabling effective bacterial eradication [182]. The Pt@V_2_C platform designed by He et al. uses the localized surface plasmon resonance phenomenon to boost photothermal conversion efficiency in the NIR-II biowindow and exhibits improved tissue penetration compared to pure V_2_C. Significantly, Pt@V_2_C exhibits dual enzyme-like activity for CDT and NIR-II-enhanced catalytic performance. The integration of Pt-based nanozymes with V_2_C nanosheets results in enhanced antibacterial efficacy and biofilm elimination properties. In practical applications, Pt@V_2_C effectively eliminates methicillin-resistant *S. aureus* from deep-seated tissues in both subcutaneous abscesses and bacterial keratitis models. Furthermore, it accelerates abscess resolution and promotes both wound and corneal healing through the synergistic effects of PTT and CDT [183].

In bacterial energy metabolism, nicotinamide adenine dinucleotide (NADH) serves as a crucial electron carrier and redox cofactor, participating in various physiological processes, including respiratory chain electron transport and the regulation of bacterial energy metabolism [184, 185]. Specifically, the depletion of NADH impairs adenosine triphosphate (ATP) synthesis and triggers the accumulation of ROS. This cascading effect leads to oxidative stress, bacterial metabolic dysfunction, and the eventual collapse of bacterial metabolic processes. The disruption of redox homeostasis ultimately results in irreversible bacterial damage, culminating in bacterial death. This mechanism underlies the antibacterial efficacy of strategies targeting bacterial NADH oxidation. Conventional approaches for light-induced NADH oxidation to NAD^+^ have predominantly relied on blue light or visible light irradiation [186], which exhibits limited tissue penetration and potential phototoxicity. NIR light stimulation presents a more advantageous alternative due to its superior tissue penetration and reduced phototoxicity. In this context, Wang et al. developed a multifunctional nanoplatform that consists of ruthenium nitrosyl (Ru-NO)-functionalized Cu and N-doped graphene quantum dots (GQDs) as a novel antibacterial agent for the effective treatment of MRSA infections and wound healing. This negatively charged nanoplatform demonstrated specific targeting to inflammatory infection sites through electrostatic interactions and exhibited high NADH dehydrogenase-like activity. Upon irradiation with 808-nm NIR light, the platform effectively photo-oxidized NADH to NAD^+^, disrupting bacterial redox balance and ultimately inducing bacterial death [187].

CuS is a semiconductor material exhibiting excellent photoelectric properties, low biological toxicity, and biodegradability. The release of Cu^2+^ from CuS can disrupt bacterial membranes and initiate GSH responses in tumor or wound microenvironments, resulting in multimodal anticancer and antibacterial effects. Moreover, as an essential trace element, copper has gained attention in wound healing and tissue engineering due to its ability to promote skin regeneration [188]. Despite these benefits, using nanozymes and photothermal agents remains hindered by limitations such as the short lifespan and limited spread of ROS and heat. Therefore, incorporating bacteria-specific recognition elements, such as antisense oligonucleotides [189] and nucleic acid aptamers, is a promising strategy. It enhances the targeted delivery and binding efficiency of antibacterial materials, enabling precise synergistic treatment. Zhang et al. developed an aptamer-functionalized, NIR light-responsive antibacterial nanoplatform (CuS@Pt-Au/Apt NPs) based on cascaded nanozymes for the synergistic treatment of wound infection in diabetic mice (Fig. 7D). The CuS@Pt-Au/Apt NPs specifically targeted bacterial surfaces through aptamer-mediated recognition. Importantly, the CuS@Pt-Au/Apt + Glu + NIR group showed the strongest bactericidal effect, with no bacterial growth on the LB plate and inhibition rates exceeding 99.99% against bacteria. The therapeutic effect was achieved through multiple mechanisms: NIR-induced hyperthermia, •OH generation via cascaded nanozymes in the high-glucose environment of diabetic infected wounds, and Cu^2+^ release from copper sulfide. These combined effects disrupted the bacterial antioxidant defense system, ultimately causing irreversible bacterial damage [190].

Light responsiveness represents a crucial advantage in the external regulation of nanozyme activity, enabling precise control and spatiotemporal selectivity [191]. However, currently reported nanozymes are predominantly metal/metal-oxide NPs or water-insoluble polymeric materials. Therefore, the development of discrete nanoarchitectures suitable for nanozyme applications in aqueous media presents significant potential. Bhattacharyya et al. reported the design and synthesis of a water-soluble Pd_12_ nanocage that exhibits strong absorption in the visible region as a potential photosensitizer. The incorporation of a benzothiadiazole photosensitizing unit in the backbone facilitated fluorescence and visible light-activated ROS generation in aqueous medium. Under white light irradiation, the nanocage demonstrated excellent OXD-like activity at low concentrations. The structure was synthesized through coordination-driven self-assembly between a benzothiadiazole-based tetra-pyridyl donor (L) and a 90° cis-blocked Pd(II) acceptor (Fig. 7E). This nanocage exhibits a Johnson polyhedral solid structural topology, featuring an unusual twisted cuboctahedron geometry. The OXD-like activity and exogenous ROS generation were successfully applied in photocatalytic antibacterial activity against MRSA. After 1 hour of light exposure, the colony photo showed a complete bactericidal effect (100%). There was no significant antibacterial activity observed in the dark, indicating that light exposure was the only external control for the activity of the nanozymes. According to the authors, this nanocage represents the first example of a water-soluble discrete coordination architecture demonstrating efficient photoinduced OXD-like nanozyme activity [192]. However, these findings need further validation in different biological environments and conditions. Additionally, it is essential to investigate the potential toxicity and long-term stability of these nanostructures in biological systems to address concerns related to practical applications. Therefore, exploring a diverse range of water-soluble nanozymes will help advance treatment strategies and fill the gaps in current research. From the perspective of stimulus-response modes, these light-activated approaches are examples of exogenously triggered systems, valued for their potential remote controllability. However, it’s a general principle that their reliance on sophisticated instrumentation often presents a barrier to clinical utility, a factor to consider [193].

#### US-responsive nanozymes

External field stimulation can greatly increase nanozyme activity. This method precisely regulates activity by coupling nanozyme-mediated and external field-driven catalysis [194]. US, which uses frequencies beyond the human auditory range, is widely used in medical diagnostics and therapeutics. Its extensive application is due to its noninvasive nature, minimal energy attenuation, and superior tissue penetration capabilities [195]. Low-intensity US overcomes the key limitation of light-based therapies: poor tissue penetration. Its noninvasive nature and deep penetration capability make it a superior alternative. Sonodynamic therapy (SDT), an emerging noninvasive therapeutic modality combining low-intensity US with sonosensitizers, has demonstrated considerable promise. Nanotechnology-enhanced SDT improves treatment efficacy while overcoming conventional limitations. This advancement enables safer and more efficient therapies [196].

US-responsive nanozymes constitute an innovative class of nanomaterials that combine the catalytic properties of enzyme-mimicking NPs with US responsiveness. The incorporation of US responsiveness in nanozyme design offers several advantages, including controlled activation, enhanced tissue penetration, and noninvasive manipulation. The mechanism of US-mediated nanozyme activation involves multiple pathways, including mechanical effects, localized heating, and cavitation phenomena. These interactions can induce structural modifications, alter reaction kinetics, or generate specific microenvironments that enhance nanozyme performance. The piezoelectric field generated by US waves serves as a controllable and noninvasive external stimulus [197, 198]. When the US, acting as an exogenous physical trigger, generates cavitation bubbles, their collapse releases intense local stress. Under this stress, piezoelectric materials instantaneously generate an internal electric field and surface piezopotential [199, 200]. This surface piezo-potential facilitates mechanical energy conversion, enabling charge carriers to traverse piezoelectric material surfaces or solution interfaces, thereby initiating various redox processes [201]. Applications of US-responsive nanozymes span diverse biomedical fields, including targeted drug delivery, cancer therapy, and antimicrobial treatments. In drug delivery systems, US can trigger the controlled release of therapeutic agents from nanozyme carriers at specific sites. Recent studies have demonstrated the efficacy of US-augmented nanozymes in infection treatment [201]. These systems can effectively absorb energy released from cavitation bubbles, inducing carrier separation and energy band bending at nanozyme surfaces, thereby enhancing their catalytic activity. Furthermore, US irradiation facilitates the formation of transient pores in biofilm matrices, improving nanozyme penetration and subsequent treatment efficacy [202, 203].

**Fig. 8:**
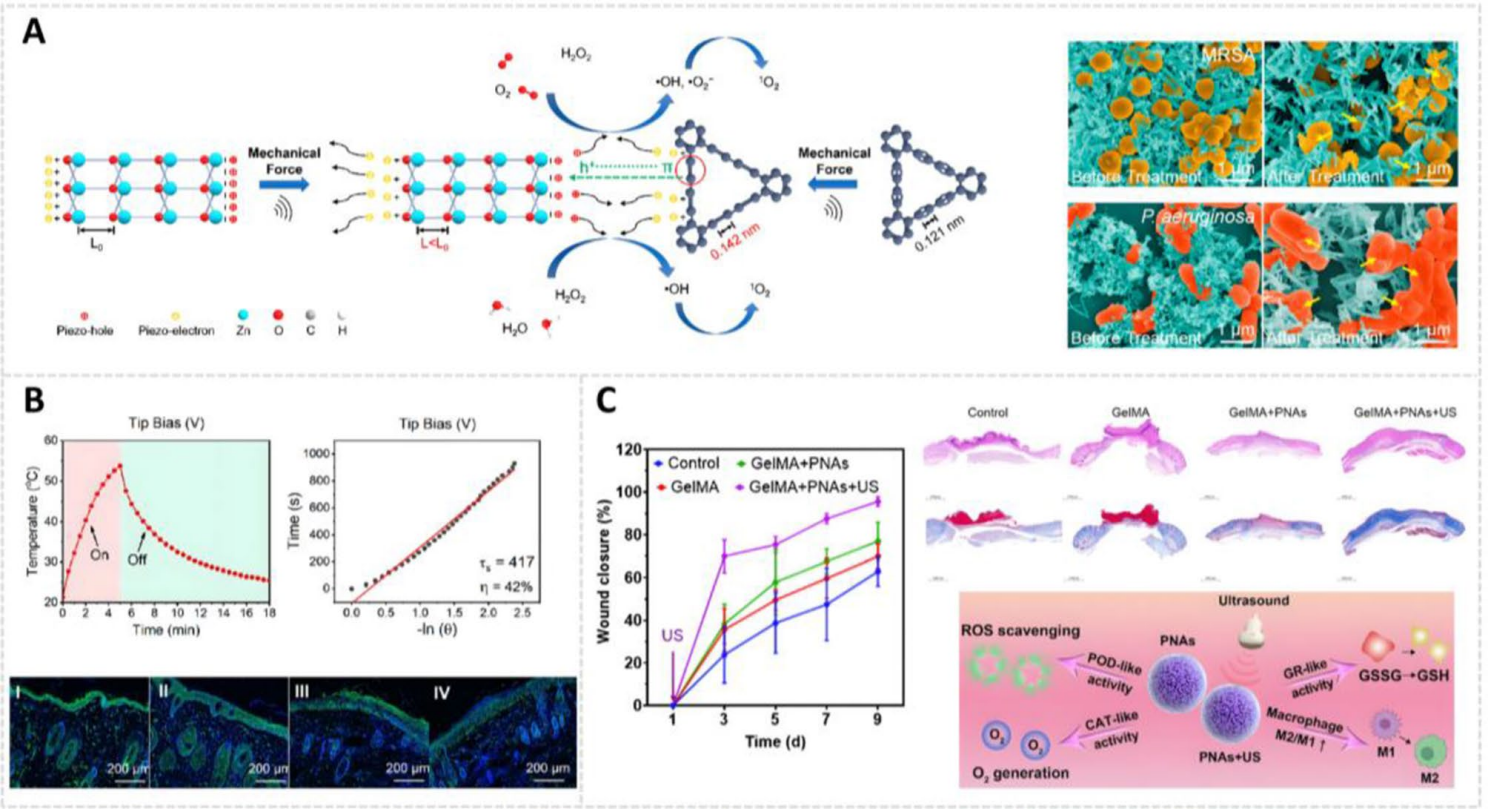
US response nanozyme in wound healing. **(A)** Schematic showing the carrier migration at the ZnO@GDY nanorod interface and the antibacterial mechanisms against MRSA and *P. aeruginosa*. SEM images demonstrate bacterial morphology distortion due to US treatment [205], Copyright 2022, American Chemical Society. **(B)** The catalytic mechanism of the Au/Cu_1.6_O/P−C_3_N_5_ system and the schematic diagram of HIF-1α immunofluorescence staining highlight the good acoustic-thermal conversion efficiency ATP, ADP, AMP as well as the oxygen supply capacity of the system [83], Copyright 2023, American Chemical Society. **(C)** The figure demonstrates the therapeutic efficacy of PNAs in accelerating diabetic wound healing in murine models while mechanistically elucidating their multifunctional enzyme-mimetic activities through ROS scavenging and macrophage polarization modulation [97], Copyright 2023, Royal Society of Chemistry

Nanozyme activity regulation remains challenging due to spontaneous activation of oxidative processes in aqueous environments, potentially causing adverse effects on healthy tissues. Therefore, there is an urgent need for strategies that can simultaneously enhance and precisely control nanozyme activity specifically in lesion areas. Graphdiyne, an emerging 2D carbon nanomaterial, exhibits unique electronic structure and geometric framework properties, making it a promising candidate for piezocatalytic nanozymes [204]. Bai et al. designed a zinc oxide nanorod@graphdiyne nanosheet (ZnO NR@GDY NS) heterostructure as a piezocatalytic nanozyme (Fig. 8A). In this design, graphdiyne functions as a conductive, adsorptive, and nanozyme catalytic layer, while ZnO serves as both a piezocatalytic and nanozyme catalytic component. This heterostructure simultaneously demonstrates intrinsic POD-like activity and strong piezoelectric responses, effectively promoting H_2_O_2_ decomposition and ROS generation under US irradiation. Moreover, this piezocatalytic nanozyme achieves nearly 100% antibacterial efficacy against multidrug-resistant pathogens, including MRSA and Pseudomonas aeruginosa, both in vitro and in vivo [205].

Advancing beyond single-enzyme systems, the integration of multi-enzymatic cascades now enables coordinated modulation of inflammation, hypoxia, and bacterial clearance in diabetic wound management [83]. Shang et al. developed a hydrogel spray system incorporating US-responsive materials for DFU therapy. This system, denoted as ACPCAH, consists of hyaluronic acid (HA)-encapsulated L-arginine, ultrasmall Au NPs, and Cu_1.6_O nanoparticle-coloaded phosphorus-doped graphitic carbon nitride nanosheets (Au/Cu_1.6_O/P-C_3_N_5_/Arg@HA). ACPCAH exhibits multiple therapeutic functions, including anti-inflammatory and antibacterial properties, O_2_ supply enhancement, and cell growth promotion for DFU treatment. Immunofluorescence staining of skin tissues indicated that the expression of HIF-1α in the ACPCAH + US group was much lower than that in the other groups, and consequently ACPCAH + US released O_2_ to ameliorate peripheral local hypoxia. The HA encapsulation serves multiple purposes: it improves nanozyme biocompatibility and stability. Additionally, it enables targeted breakdown by hyaluronidase in biofilms. This facilitates the release of L-arginine and nanozymes, enhancing their interaction with bacteria. The acousto-thermal conversion efficiency (η) of Au/Cu_1.6_O/P-C_3_N_5_ was 42%, enabling it to initiate an SOD-CAT-GOx-POD/NOS pentaenzyme-like cascade reaction (Fig. 8B). This cascade effectively reduces inflammation, alleviates hypoxia, decreases blood glucose levels, promotes angiogenesis, and eliminates pathogenic bacteria. These combined effects accelerate diabetic wound healing [83].

GSH, an endogenous antioxidant, serves as the body’s natural reducing agent and is mainly synthesized through GSH reductase under the catalytic influence of NADPH [206]. Platinum, as a desirable heterogeneous hydrogenation catalyst, facilitates hydrogen atom transfer to carbon p orbitals through surface activation, thereby effectuating hydrogenation reactions [207]. Additionally, nanozymes composed of platinum NPs or their assemblies have demonstrated antioxidant enzymatic activities, including CAT and POD, which contribute to ROS scavenging. Zhang et al. developed a comprehensive therapeutic strategy based on platinum nanozymes (PNA) (Fig. 8C). They synthesized a hybrid material from dynamic covalently bonded metal-organic coordination polymers. This material was used as a template to fabricate densely packed assemblies of platinum nanoparticles. This compact assembly exhibits multiple functionalities: it effectively mimics antioxidant enzymes (CAT, POD) and, under US stimulation, enhances electron polarization through the surface plasmon resonance effect. Notably, this system demonstrates GSH reductase-like activity, promoting GSH generation [208]. By mimicking CAT and POD activities, they effectively catalyze H_2_O_2_ decomposition, alleviating hypoxia, while simultaneously generating GSH under US stimulation to enhance ROS scavenging. Wound healing images, closure traces, and quantitative analyses at different time points after various treatments show that PNA is especially effective in accelerating diabetic wound healing when combined with US. Hematoxylin-eosin images further demonstrate that PNA enhances diabetic wound healing by promoting the formation of both the epidermis and dermis. The system shows remarkable biological effects, including restoration of fibroblast and endothelial cell proliferation, enhanced keratinocyte migration, and promotion of macrophage polarization toward the M2 phenotype. These combined effects lead to potent anti-inflammatory responses and accelerated healing of infected wounds [97]. Although research on US-responsive nanozymes shows enormous potential for therapeutic applications, the variability in experimental conditions requires further investigation. For instance, the use of different types of US stimuli may lead to inconsistent results, highlighting the need for standardized experimental protocols. Addressing these research gaps is crucial for the successful clinical translation of US-responsive nanozymes.

### Chemical and biochemical stimuli-responsive nanozymes

#### pH-responsive nanozymes

pH-responsive nanozymes exhibit enzyme-like catalytic activities modulated by environmental pH variations. These pH-responsive nanozymes are designed to change their structure and electronic properties with varying pH, which in turn affects their catalytic activity. The pH-dependent responsiveness of these nanozymes enables their selective activation in distinct biological microenvironments. For instance, they can be specifically activated in the acidic microenvironment of tumors or tissues, ensuring enhanced targeting efficiency and minimized off-target effects. Furthermore, the multi-enzymatic activities of the biomaterial suggest a more varied treatment approach and can be adjusted based on the pH of the wound, allowing for combined therapeutic effects at various stages of healing. For example, Zhang et al. constructed an FeOOH@Fe-Serine@Au nanosystem that possesses both GOx and POD activities. The pH-dependent enzymatic activities of GOx and POD in this biomaterial show that at weakly acidic conditions (pH = 5), POD activity predominates, whereas at neutral to weakly alkaline environments (pH = 7–9), GOx activity predominates [209].

**Fig. 9:**
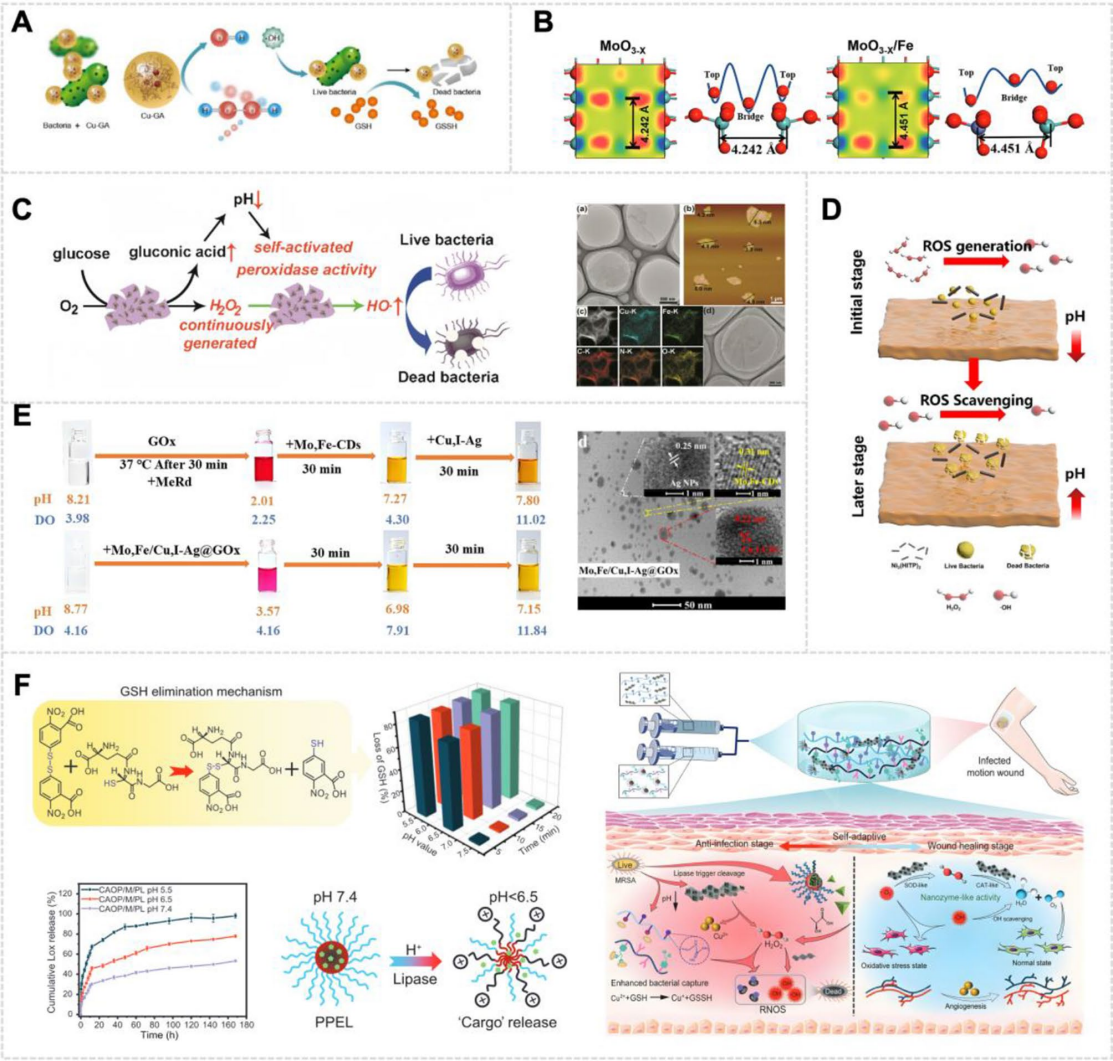
pH-responsive nanozymes for wound healing. **(A)** Diagram illustrating the antibacterial action of Cu-GA nanorods against bacterial infections, highlighting their ability to reduce GSH levels and produce ROS [220], Copyright 2023, Elsevier. **(B)** Electron density difference maps showing how varying environments affect the catalytic mechanisms of the Fe-doped MoO_3_−X nanozyme, enhancing its antibacterial and O_2_-generating capabilities [210], Copyright 2023, John Wiley and Sons Ltd. **(C)** Schematic and images depicting the self-activated POD activity of the 2D MOF/GOx nanocatalyst, illustrating its mechanism in generating H_2_O_2_ from glucose to kill bacteria [167], Copyright 2019, American Chemical Society. **(D)** Illustration of the switching roles of nanozymes: initially generating ROS in an acidic environment, transitioning to ROS scavenging in a neutral environment, improving wound healing outcomes [89], Copyright 2024, Academic Press Inc. **(E)** Chart summarizing the effects of various treatments on GSH levels across different pH conditions, along with imaging showing the structural details of the nanozyme interactions [226], Copyright 2024, Elsevier. **(F)** Schematic showing the self-adaptive mechanism of the hydrogel: enhanced bacterial capture, controlled release of therapeutic agents, and promotion of wound healing through oxidative stress modulation and angiogenesis [228], Copyright 2024, Oxford University Press

The pH environment plays a crucial role in regulating both enzyme-like behavior and O_2_ delivery to damaged tissues [5]. Yu et al. developed a bifunctional nanozyme system, termed ADFM, consisting of atomically dispersed Fe-doped oxygen-deficient molybdenum oxide (MoO_3_-x) (Fig. 9B). This nanozyme demonstrates enhanced CAT-like activity and inherent POD-like activity. It helps decompose H_2_O_2_ into both strongly oxidizing •OH for bacterial infection control and O_2_ for tissue regeneration. The therapeutic mechanism of ADFM demonstrates pH-dependent behavior. In the initial acidic wound environment, ADFM predominantly exhibits POD-like activity, achieving effective antibacterial function. As the wound pH gradually increases, the CAT-like activity becomes dominant, addressing tissue hypoxia. The nanozyme’s excellent features help wounds heal quickly in several ways: breaking down bacterial biofilms, damaging their genetic material, reducing inflammation, and producing O_2_ to aid tissue repair [210].

Bacteria colonizing infection sites form biofilms [211], which cause anaerobic metabolism and the production of acids like lactic acid, malic acid, and acetic acid [212]. This metabolic activity results in reduced pH values within the wound microenvironment. Notably, in cases of MRSA infection, the local pH may decrease to approximately 5.5 or even lower [213]. Stimuli-responsive biomaterials can deliver on-demand antibacterial effects in two main ways: by releasing bactericidal components or altering their properties to respond to external or internal stimuli [214]. While external stimuli offer greater controllability, their therapeutic application often occurs after severe infection has developed, potentially increasing patient discomfort [215]. In contrast, endogenous stimuli-responsive materials can respond rapidly to changes in the wound microenvironment, enabling more timely and effective infection control [216].

While copper-containing nanozymes have emerged as promising antibacterial materials, their effectiveness is significantly limited by elevated GSH levels in bacterial microenvironments [217]. To address this challenge, multi-enzyme systems have been proposed, as GSH-Px effectively reduces GSH levels, while its combination with POD can simultaneously increase ROS levels and consume excess GSH [218]. Additionally, gallic acid (GA), a natural polyphenol possessing antioxidant properties, displays SOD activity that can mitigate inflammation and oxidative stress-induced damage [219]. Tian et al. developed Cu-GA nanorods featuring triple enzyme-like activities (POD, GSH-Px, and SOD) for effective treatment of bacterial infections (Fig. 9A). Notably, Cu-GA exhibits pH-dependent dual functionality: generating substantial ROS under acidic conditions while scavenging ROS in neutral environments [220].

MOF-based POD mimics encounter two primary challenges that restrict their biomedical application in vivo: they require strong acidity (pH 3–4) for optimal enzyme-like activity, and traditional systems depend on hazardous concentrations of H_2_O_2_ to generate •OH radicals, potentially damaging healthy tissue [221, 222]. To address these limitations, Liu et al. developed an innovative MOF-based hybrid nanocatalyst as a biocompatible, self-activating cascade system. They selected an ultrathin 2D MOF nanosheet (2D Cu-TCPP(Fe)) as the POD mimic platform (Fig. 9C) [167]. This platform was functionalized by immobilizing GOx to create the hybrid nanocatalyst. The system operates through a cascade mechanism: GOx continuously converts non-toxic glucose into gluconic acid and H_2_O_2_, eliminating the need for direct H_2_O_2_ administration. The generated gluconic acid creates a localized pH reduction from 7 to 3–4, optimally activating the POD-like activity of the 2D Cu-TCPP(Fe) nanosheets. The in situ produced H_2_O_2_ then undergoes catalysis by the activated nanosheets, resulting in efficient •OH generation for enhanced antibacterial activity. In mouse wound models, the nanocatalyst effectively utilizes endogenous glucose to initiate the cascade reaction: lowering local pH, activating POD-like activity, and generating bactericidal •OH species, ultimately achieving efficient bacterial elimination [223, 224].

The inflammatory response plays a crucial role in wound healing by facilitating the clearance of necrotic material and promoting the release of growth factors [225]. However, clinical studies have demonstrated that chronic wounds are often associated with excessive and persistent inflammation. Thus, modulating the inflammatory response has emerged as a key therapeutic strategy for accelerating wound healing, indicating the importance of therapeutic agents capable of regulating inflammation at different treatment stages. During bacterial infection, the local tissue microenvironment becomes acidic. Chen et al. developed a pH-responsive nickel-based MOF (Ni-MOF) featuring dual enzyme-like activities (Fig. 9D). This novel Ni-MOF, incorporating POD-mimetic Ni-N_4_ units and secondary amines, demonstrates pH-dependent catalytic behavior. The therapeutic mechanism of Ni-MOF exhibits distinct pH-dependent phases. In the acidic environment of bacterial infections, this material produces ROS. This process both kills bacteria and promotes pro-inflammatory macrophage activity, thereby boosting the immune response. When biofilms are removed and the local pH becomes neutral, the Ni-MOF switches to SOD-like behavior. It reduces ROS levels and promotes anti-inflammatory macrophage polarization (M2 phenotype). This adaptive nanozyme system manages infection control and immune response by balancing ROS production and removal based on the changing environment during treatment [89].

To address the dual challenges of hyperglycemia and bacterial infection in wound healing, researchers have developed glucose-activated cascade reactions based on GOx and POD [112]. In this system, GOx catalyzes glucose oxidation in the presence of O_2_, generating H_2_O_2_ and gluconic acid. Subsequently, POD enzymes convert H_2_O_2_ to •OH, simultaneously achieving glucose reduction and bacterial elimination [167]. This cascade process effectively reduces glucose levels while avoiding direct H_2_O_2_ administration and creates an acidic microenvironment favorable for POD activity. However, two critical challenges persist in implementing effective cascade reactions: the impact of pH variations on nanozyme activity and the O_2_-dependent nature of glucose oxidation. Furthermore, conventional nanozymes often exhibit limited catalytic efficiency and poor synergistic multi-enzyme activities, restricting their application in diabetic wound healing. To address these limitations, Li et al. developed an innovative nanocomposite (Mo, Fe/Cu, I-Ag@GOx) featuring multiple enzyme-like activities, which was incorporated into a multifunctional fluorescent hydrogel (Fig. 9E). This system employs two mechanisms based on wound conditions. First, GOx catalyzes the conversion of glucose and O_2_ into gluconic acid and H_2_O_2_, which are subsequently converted into O_2_^•−^ and •OH to kill bacteria. When the wound becomes alkaline, a distinct reaction pathway is initiated: the gel breaks down O_2_^•−^ into O_2_ and H_2_O_2_, then converts the H_2_O_2_ into additional O_2_. This dual system reduces oxidative stress and improves O_2_ levels in the wound [226].

Intricate bacterial infections require novel therapies. Autonomous intelligent wound dressings capable of detecting and addressing bacterial environments would be optimal [227]. Yang et al. developed a sophisticated hydrogel platform incorporating two key components: a pH-responsive H_2_O_2_ self-supplying composite nanozyme (MSCO) and pH/enzyme-sensitive, bacteria-responsive triblock micelles loaded with lactate oxidase (PPEL) (Fig. 9F). These components were integrated into a hydrogel matrix composed of L-arginine-modified chitosan and phenylboronic acid-modified oxidized dextran. The therapeutic mechanism of this system proceeds through multiple coordinated steps. In bacterial infection-induced acidic environments, the hydrogel network undergoes structural collapse, triggering the release of PPEL micelles and MSCO nanozyme. The PPEL micelles respond to acidic metabolites and lipase by releasing lactate oxidase, which catalyzes lactic acid decomposition to generate H_2_O_2_. This locally produced H_2_O_2_ then synergizes with the MSCO nanozyme to convert L-arginine from chitosan into NO. The antibacterial action involves several concurrent mechanisms: H_2_O_2_ enhances the POD-like activity of MoS_2_, thereby amplifying the Cu^2+^-catalyzed Fenton reaction to generate ROS. The resulting ROS, working in concert with NO, effectively eliminates bacteria. Additionally, MSCO releases Cu^2+^ ions that deplete bacterial GSH, compromising bacterial defense against oxidative stress. In mildly alkaline wound environments, the hydrogel system demonstrates efficient oxidative stress regulation through multiple nanozyme activities. Furthermore, the gradual release of Cu^2+^ from MSCO hydrolysis, combined with L-arginine, promotes wound angiogenesis [228]. While many studies emphasize the effectiveness of pH-responsive nanozymes in regulating local pH levels and enhancing enzyme activity, reports on their performance vary across different experimental setups. The relationship between pH changes and enzyme activity may differ depending on the specific microenvironment.

#### ATP-responsive nanozymes

ATP-responsive nanozymes are advanced catalytic materials. They combine traditional nanozyme properties with the ability to respond specifically to ATP, which is a key energy carrier in biology. These hybrid systems combine two important aspects: the powerful catalytic ability of nanozymes and their ATP-dependent selective activation. This combination allows for precise regulation of their activity within intricate biological contexts [229]. The architectural design of ATP-responsive nanozymes involves the strategic incorporation of ATP-sensitive moieties into the nanozyme framework. These specialized components undergo specific structural or chemical modifications upon ATP recognition, leading to controlled modulation of the nanozyme’s catalytic properties. This sophisticated response mechanism enables intelligent regulation of enzymatic activity based on local ATP concentrations, providing a level of spatial and temporal control previously unattainable with conventional nanozyme systems [230]. The ATP-dependent functionality makes these materials particularly valuable for diverse biomedical applications requiring precise activity modulation in response to cellular energy states.

**Fig. 10:**
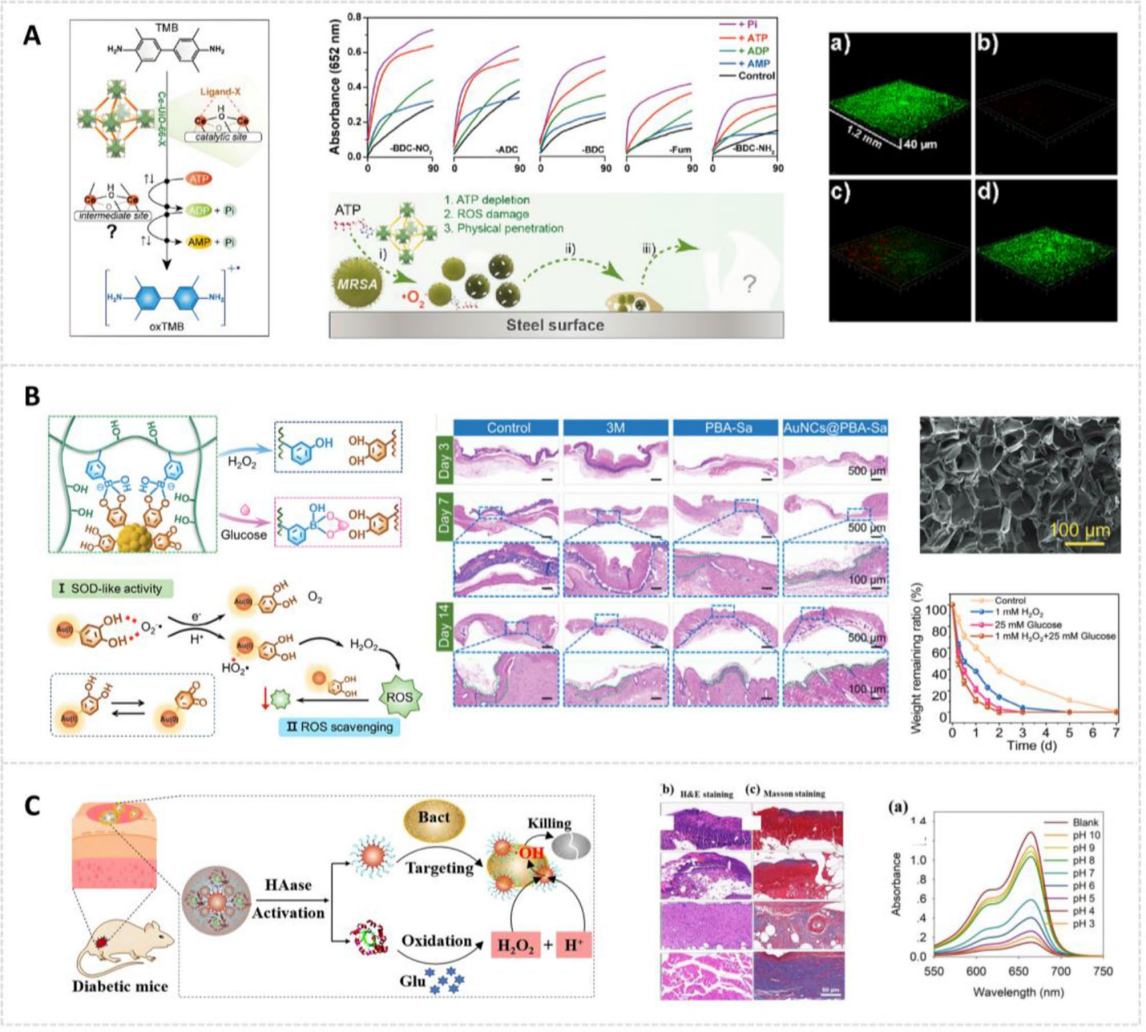
ATP, redox, and microenvironment-responsive nanozymes. **(A)** Mechanism of Ce-UiO-66-X nanozymes: ATP-mediated activation of OXD-like activity and antimicrobial effect [232], Copyright 2023, Elsevier. **(B)** Dual-responsive behavior of AuNCs@PBA-Sa hydrogel: ROS/glucose-triggered release mechanism and in vivo diabetic wound treatment efficacy [237], Copyright 2024, John Wiley and Sons Ltd. **(C)** APGH-mediated wound healing process: mechanism of chemodynamic sterilization, in vivo treatment outcomes, and POD-like activity characterization [113], Copyright 2024, John Wiley and Sons Ltd

Extracellular ATP represents a promising biomarker for guiding targeted therapeutic interventions [229]. Huang et al. demonstrated the isoreticular modulation of dual enzyme-mimicking activities in Ce-UiO-66-X through systematic linker substitution with varying electron-withdrawing capabilities [231]. The researchers employed an isoreticular design principle in nanozyme development to establish a specific domino biomimetic reaction targeting drug-resistant bacterial biofilms (Fig. 10A). A series of isoreticular MOFs, designated as Ce-UiO-66-X (where X represents different organic linkers: BDC, BDC-CH_3_, BDC-OH, BDC-NH_2_, BDC-NO_2_, ADC, or Fum), were synthesized to exhibit linker-dependent dual enzymatic activities mimicking hydrolytic apyrase and redox OXD. The therapeutic mechanism involves a cascade process: the phosphorous-containing products (ATP/ADP/AMP/Pi) generated through ATP hydrolysis spontaneously activate redox reactions within Ce-UiO-66-X. The results indicate that all ATP, ADP, AMP, and Pi species can act as nanozyme activators, accelerating the OXD mimetic reaction of Ce-UiO-66-X. This sequential activation leads to multiple antibacterial effects: inhibition of microbial metabolism, prevention of initial bacterial adhesion, and destruction of MRSA cells. Additionally, biofilm growth was assessed using live/dead staining and confocal laser scanning microscopy. The results revealed that the Ce-UiO-X nanozyme displayed linker-dependent anti-biofilm activity, attributed to the isoreticular engineered domino nanozyme reaction. Overall, these findings emphasize the possible use of Ce-UiO-X nanozymes as effective agents against bacterial infections [232]. Research indicates that these nanozymes effectively target resistant bacterial biofilms and regulate bacterial adhesion. Nonetheless, the interactions between these entities and other biological components, such as proteins and signaling molecules, remain inadequately understood. Exploring these interactions is important for making ATP-responsive nanozymes more effective and widening their clinical use.

#### ROS-responsive nanozymes

ROS-responsive nanozymes are smart catalytic materials that activate specifically in disease sites with abnormal ROS levels [85, 144]. When exposed to high ROS concentrations, these materials undergo structural or functional changes to either release drugs, neutralize excess ROS, or trigger therapeutic reactions. This targeted activation focuses treatment precisely on diseased areas while protecting healthy tissues [233, 234].

Gold nanoclusters (AuNCs) are a novel type of nanomaterial with several distinguishing characteristics. They are small (< 2 nm), biocompatible, provide multiple therapeutic options, and have excellent pharmacokinetic properties. These characteristics make them ideal for developing advanced biomedical platforms [235]. AuNCs have precise structure-property relationships, allowing for controlled functionalization via various ligands. This process produces properties such as antibacterial activity, antioxidant capacity, enzyme-like behavior, and immunomodulatory effects [236]. Wang et al. developed marine-derived multifunctional AuNCs that serve dual roles in hydrogel fabrication: acting as both gelation crosslinkers and bioactive components (Fig. 10B). The incorporation of marine mussel-derived catechol ligands (L-3,4-dihydroxyphenylalanine) enhances the SOD-mimetic activity of the AuNCs. Uniform crosslinking of sodium alginate (PBA-Sa) by AuNCs forms AuNCs@PBA-Sa hydrogels. These hydrogels exhibit remarkable self-healing capability, controllable degradation rates, and facile removability. Furthermore, the incorporation of AuNCs significantly enhances the mechanical properties and confers favorable tissue adhesion for rapid hemostasis. In response to the pathological characteristics of diabetic wounds, specifically elevated ROS and glucose levels, the hydrogel demonstrates intelligent adaptability through the selective breakage of boronate ester bonds between AuNCs and PBA-Sa. The subsequently released AuNCs function as clusterzymes (enzyme-mimicking nanoclusters), effectively promoting M2-macrophage polarization and demonstrating significant anti-inflammatory and pro-regenerative effects. As shown in a representative image of hematoxylin-eosin-stained wound tissue, after 14 days, the AuNCs@PBA-Sa hydrogel group exhibited completely healed skin tissue, characterized by a mature thin epidermis (thickness: 17.4 μm), regenerated blood vessels, and newly formed hair follicles. In contrast, the Control and 3M groups displayed an undesirably thick epidermis and immature skin tissues, indicating limited wound healing efficiency [237].

### Microenvironment-responsive nanozymes

Advanced nanozyme systems exhibit significant adaptability and potent therapeutic effects in bacterial infections via multiple mechanisms [238]. These nanozymes can be engineered to interact with bacterial hyaluronidase, facilitating targeted activation and regulated drug release in the context of infections. Their effectiveness is augmented by their interaction with the infection environment. For example, in diabetic wounds, they can utilize elevated glucose concentrations to generate H_2_O_2_in situ, thereby amplifying their antibacterial efficacy through CDT. The incorporation of aptamer-based recognition elements further enhances their specificity for pathogenic bacteria [239]. These nanozyme systems adjust the local environment by optimizing pH levels to enhance enzyme activity. This helps overcome physiological barriers that limit traditional antimicrobial agents’ effectiveness [115]. This adaptive functionality represents a significant advancement over traditional therapeutic approaches. However, while endogenous response systems are better suited for dynamic environments, they face control limitations due to biomarker variability.

Nanozymes exhibit optimal POD-like activity in acidic conditions (pH 3–6) through H^+^ preabsorption and base-catalyzed H_2_O_2_ decomposition [115]. However, most physiological environments maintain alkaline conditions (pH > 8.0 in some chronic wound tissues), except for tumor microenvironments [166], which presents a significant limitation for nanozyme functionality [240]. Additionally, physiological H_2_O_2_ concentrations are typically insufficient to achieve effective CDT [241]. To address these physiological constraints regarding pH and H_2_O_2_ availability, Chen et al. developed an activatable targeted nanozyme system for CDT applications (Fig. 10C). Their design incorporated aptamer-functionalized platinum nanozymes (Apt-PtNZ) and GOx co-encapsulated within an HA shell, forming a nanocapsule designated as “APGH”. This system was specifically designed for CDT applications in diabetic wounds, which characteristically present elevated glucose levels and alkaline pH conditions [239]. The therapeutic mechanism proceeds through multiple sequential steps: Initially, bacterial hyaluronidase triggers the degradation of the HA shell [242], enabling the controlled release of Apt-PtNZs and GOx. The released Apt-PtNZs specifically target bacteria through aptamer-mediated recognition [243], while GOx catalyzes the conversion of glucose to gluconic acid and H_2_O_2_. This dual-action mechanism serves two purposes: it creates a localized acidic environment that enhances POD-like activity while simultaneously generating H_2_O_2_ for the in situ production of •OH at bacterial surfaces. This infection-responsive design enables self-activated and enhanced therapeutic efficacy, resulting in efficient chemodynamic bacterial elimination under physiological conditions [113].

### Multi-stimuli-responsive nanozymes

#### Dual-modal systems

##### pH and NIR light-responsive nanozymes

**Fig. 11:**
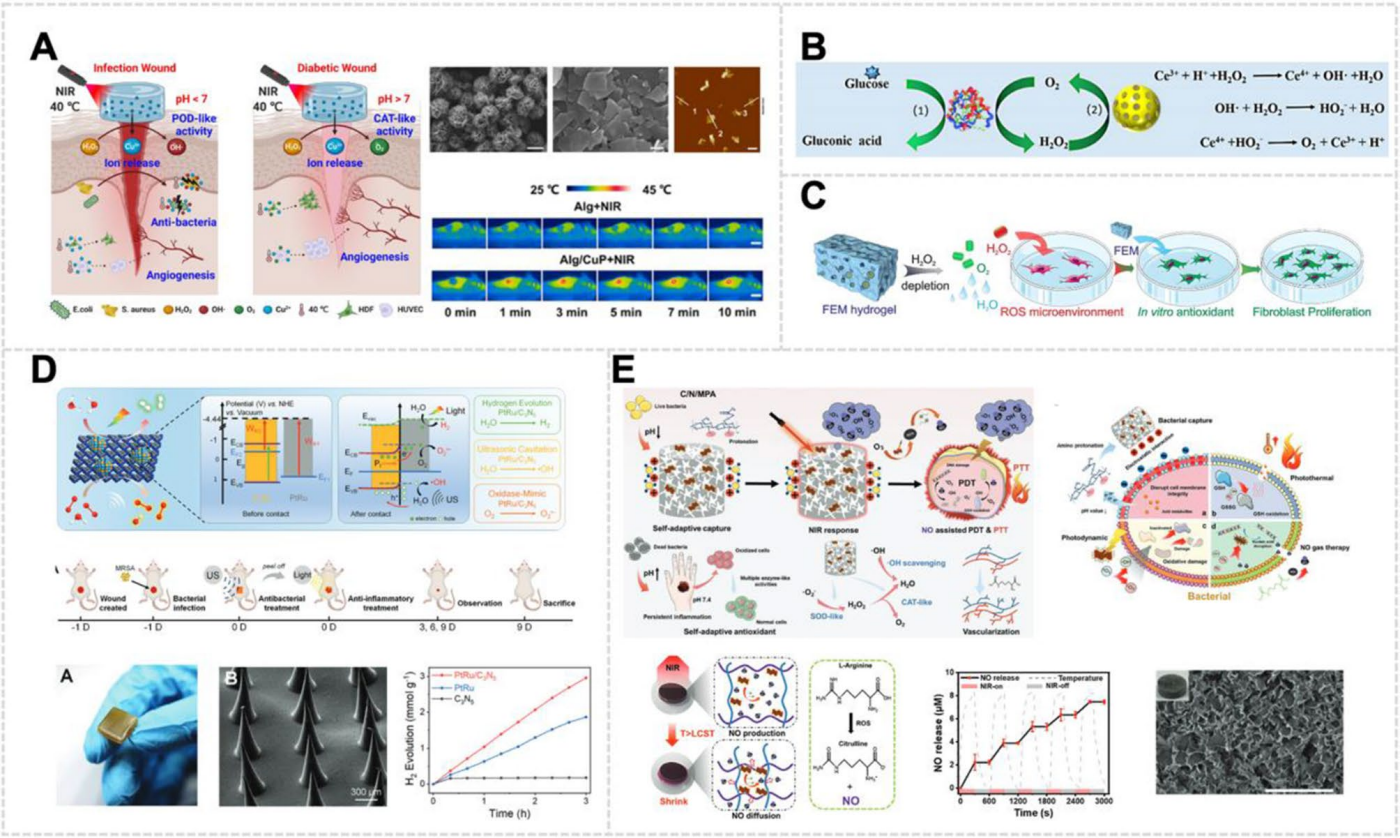
Multi-stimuli responsive nanozymes. **(A)** Schematic of pH and NIR light-responsive CuP composite hydrogel and its pH-dependent and NIR-activated antibacterial and angiogenic properties [246], Copyright 2023, American Chemical Society. **(B)** Schematic illustration of the ROS and glucose-responsive Ce-driven nanozyme for diabetic wound healing: mechanism of glucose conversion for wound microenvironment regulation [81], Copyright 2022, Elsevier. **(C)** The diagram demonstrates the FEM hydrogel’s role in ROS depletion and its effects on fibroblast proliferation [155], Copyright 2020, American Chemical Society. **(D)** NIR and US-responsive nanozyme PtRu/C_3_N_5_ nanozyme’s catalytic mechanism and wound healing strategy in MRSA-infected model [201], Copyright 2023, John Wiley and Sons Ltd. **(E)** Multi-responsive cryogel with temperature, pH, and NIR responsiveness, exhibiting antibacterial, angiogenic, and controlled NO release properties [101], Copyright 2023, John Wiley and Sons Ltd

Nanoparticles exhibit pH-dependent enzymatic activities, demonstrating POD or OXD activities under acidic conditions, while displaying SOD or CAT activities in alkaline environments [244]. This adaptability allows these nanoparticles to dynamically respond to changes in pathological microenvironments, optimizing ROS regulation [245]. Furthermore, Cu ions promote wound healing by stimulating endothelial cell proliferation and migration while upregulating angiogenic gene expression, including HIF-1α and VEGF. Feng et al. developed an advanced wound dressing that integrates conventional alginate hydrogel (Alg) with biodegradable copper hydrogen phosphate (CuP) nanozymes (Fig. 11A). In alkaline conditions typical of diabetic wounds, the Alg/CuP dressing promotes angiogenesis and accelerates healing through its CAT-like activity, which converts H_2_O_2_ to molecular O_2_. This effect is complemented by the controlled release of Cu ions. When bacterial infection induces acidification of the wound environment, the material’s catalytic behavior shifts to POD-like activity. This activity generates •OH, which synergistically works with copper ions to eliminate bacteria and biofilms. Furthermore, NIR irradiation-induced mild hyperthermia enhances both the catalytic activities of the nanoparticles and the bioactivity of Cu ions, thereby promoting the healing of infected and diabetic wounds [246].

##### ROS and glucose-responsive nanozymes

Diabetic wounds are characterized by a complex pathological microenvironment featuring hyperglycemia, hypoxia, and elevated ROS levels. Effective regulation of this multifaceted microenvironment presents a promising therapeutic strategy for enhancing chronic diabetic wound healing outcomes. Yu et al. developed an innovative nanozyme system that is responsive to glucose and ROS. This system utilizes cerium-driven coassembly with a dual-ligand approach, incorporating alendronic acid and 2-methylimidazole, along with GOx (Fig. 11B). This engineered nanozyme system exhibits both SOD and CAT mimetic activities, enabling effective modulation of excessive ROS levels. Notably, the system demonstrates the capability to catalyze the conversion of excess H_2_O_2_, generated during glucose oxidation, into O_2_. This dual-function mechanism helps maintain O_2_ homeostasis in the wound environment and reduces the cytotoxic effects associated with GOx activity. As a result, it facilitates synergistic repair of diabetic wounds [81].

##### pH and ROS-responsive nanozymes

Diabetic skin is characterized by an acidic and oxidative microenvironment [247]. MnO_2_ nanosheets, as emerging nanozymes, demonstrate the capability to catalyze the decomposition of endogenous H_2_O_2_ into O_2_, offering a promising approach for mitigating oxidative stress and hypoxia [248]. Wang et al. engineered an injectable multifunctional hydrogel (FEMI) to address multiple challenges in diabetic wound healing, including multidrug-resistant bacterial infections, hyperglycemia, and oxidative stress (Fig. 11C). The FEMI hydrogel was synthesized via Schiff-base reactions between ε-polylysine-coated MnO_2_ nanosheets (EM) and insulin-loaded self-assembled aldehyde Pluronic F127 micelles. In the acidic diabetic wound environment, the Schiff-base crosslinking networks undergo degradation, while the MnO_2_ nanozymes actively modulate the hostile oxidative microenvironment through H_2_O_2_ decomposition to O_2_, thereby alleviating oxidative stress. Under acidic and oxidative conditions (100 µM H_2_O_2_, pH 6.5), the hydrogel exhibited enhanced insulin release kinetics. The release rate increased 3.19-fold in the first 6 h compared to control conditions. This pH/redox dual-responsive FEMI hydrogel system enabled spatiotemporal control of insulin release, effectively regulating blood glucose in the acidic and oxidative microenvironment of diabetic wounds [155].

##### NIR light and US-responsive nanozymes

OXD-like nanozymes can convert O_2_ molecules at infection sites into antimicrobial ROS. They are promising candidates for nanozyme-assisted bacterial therapy. However, these intrinsic OXD-like nanozymes typically exhibit limited antibacterial efficacy, making the enhancement of their therapeutic performance a critical research focus [249, 250]. Furthermore, continuously active nanozymes may induce undesired effects on healthy cells while compromising their antibacterial effectiveness [251]. 2D porous C_3_N_5_ nanosheets, known for their piezoelectric properties [252], demonstrate US-enhanced nanozyme activity. Shi et al. developed an advanced multifunctional heterostructure combining ultrasmall platinum-ruthenium (PtRu) nanoalloys with porous graphitic carbon nitride (C_3_N_5_) nanosheets (Fig. 11D). This composite system exhibits dual functionality: US-enhanced OXD-mimetic nanozyme activity and photocatalytic hydrogen generation capability. The PtRu nanoalloys serve as photocatalysts for H₂ production, supporting anti-inflammatory therapy [253]. The formation of Schottky junctions promotes charge separation and piezoelectric field generation, simultaneously enhancing nanozyme activity and hydrogen production efficiency. This design enables precise control of antibacterial and anti-inflammatory functions through distinct external stimuli: US and light irradiation [201].

#### Multimodal systems

##### Temperature, pH, and NIR light-responsive nanozymes

Carboxymethyl chitosan (CMCS) wound dressings trap bacteria by using electrical charges from their amino groups to stick to the bacteria’s outer layer [254]. This effectively confines bacteria within the ROS-mediated damage zone. Additionally, MoS_2_ exhibits excellent photocatalytic properties, enabling both photodynamic therapy and PTT for bacterial elimination [255]. However, photocatalytic antibacterial efficacy alone remains insufficient for comprehensive infection treatment. Gas therapy has emerged as a promising wound healing approach due to its rapid cellular permeability and ability to enhance microcirculation [256]. Among various therapeutic gases, NO demonstrates unique advantages in bacterial infection inhibition and biofilm elimination. Nanozymes can exhibit differential enzyme-like catalytic activities in response to varying wound healing microenvironments [40]. In neutral wound environments, MoS₂ exhibits both SOD and CAT-like activities: SOD-like activity catalyzes O_2_^•−^ conversion to H_2_O_2_ and O_2_, while CAT-like activity facilitates the decomposition of both endogenous and exogenous H_2_O_2_ into O_2_ [257]. This adaptive nanozyme activity minimizes ROS loss during photodynamic antibacterial treatment while scavenging excess ROS during post-infection healing [136]. Yang et al. developed self-adaptive wound dressings with multiple stimuli-responsive properties. They designed MoS_2_ nanocarriers loaded with ROS-responsive NO precursor L-arginine (MSPA) and incorporated them into carboxymethyl chitosan/poly(N-isopropylacrylamide) (CMCS/PNIPAM) cryogels (Fig. 11E). This composite system responds to multiple stimuli (pH, NIR, and temperature) to address different stages of infected wound healing. In acidic environments of bacterial infection, CMCS amino groups self-adaptively protonate, enhancing bacterial capture through electrostatic interactions. Under NIR irradiation, MSPA generates ROS to promote cascade NO release, providing synergistic photodynamic and photothermal antibacterial effects. Following infection elimination, as wound pH increases to approximately 7.4, MSPA exhibits SOD and CAT-like activities, effectively eliminating excess free radicals and mitigating oxidative stress [101].

## Perspectives and challenges

Nanozymes exhibit enzyme-like activities, stability, and targeted delivery capabilities. They are promising tools for wound healing and act as effective catalysts for biochemical reactions. Exogenous stimuli, such as light, US, electric fields, and magnetic fields, can regulate nanozyme activity without altering its intrinsic properties (Table 1) [258]. Although remarkable advances and significant progress in responsive nanozymes in biomedicine have been achieved, this field is still in its nascent stage, and there remain challenges to be overcome in the development and clinical translation of nanozymes. These include ensuring biosafety and facilitating clinical translation, optimizing specificity, improving the limited understanding of the biological mechanism, and enhancing reproducibility and scalability.

**Table 1:** Examples of responsive nano-enzymes for promoting wound healing

Stimuli-Responsive	Enzyme activity	Chemical Formula	Morphology	Function	Form	References
NIR Light-Responsive	POD	MoWS_2_	Layered nanosheets	Antibacterial	Suspension	[175]
	POD	CeCu-MOFCe_0.5_	2D nanosheets	Antibacterial	Water/ethanol solution	[179]
	OXD, POD	Pt@V_2_C	nanosystem	Antibacterial	Phosphate Buffer Solution	[183]
	NADH dehydrogenase	Cu, N-GQDs@Ru-NO	2D disk-like morphology nanoplatform	Antibacterial	Phosphate Buffer Solution	[187]
	OXD, POD	CuS@Pt-Au/Apt nanoparticles	Spherical morphology nanoparticles	Antibacterial	Aqueous solution	[190]
	OXD	Pd_12_ nanocage	Twisted cuboctahedron geometry-based nanocage	Antibacterial	Aqueous solution	[192]
US-Responsive	POD	ZnO@GDY nanorods	Nanorods	Antibacterial	Skin patch	[205]
	SOD, CAT, GOx, POD, NOS	ACPCAH	Nanosheets	Anti-inflammatory Antihypoxic Hypoglycemic Proangiogenic Bactericidal	Hydrogel spray	[83]
	CAT, POD, GR	PNAs	Nanospheres	Antihypoxic Antioxidant GSH-generating Anti-inflammatory Re-epithelialisation Fibroblast proliferation Immunomodulation	Hydrogel	[97]
pH-Responsive	Acidic: POD, GSH-Px Neutral: SOD	Cu-GA	Amorphous structural nanorods	Antibacterial Antioxidant Proangiogenic	Solution	[220]
	Acidic: POD Neutral: CAT	ADFM	Nanoparticles	Antibacterial Anti-inflammatory	Aqueous solution	[210]
	Neutral: GOx Acidic: POD	2D Cu-TCPP(Fe)/GOx	Ultrathin 2D nanosheets	Antibacterial	Band-aid dressing	[167]
	Neutral: Antioxidant enzymes Acidic: POD	Cu ND/CG	Small dimensions dot-like particles	Antibacterial Proangiogenic Anti-inflammatory	CG Hydrogel	[91]
	Acidic: POD Neutral: SOD	Ni_3_(HITP)_2_	Nanorods	Antibacterial Immunomodulation	Solid Powder	[89]
	Neutral: GOx, POD, OXD, CAT Alkaline: SOD	Mo, Fe/Cu, I-Ag@GOx	Monodisperse spherical-structured nanoparticles	Proangiogenic Antibacterial Re-epithelialisation	Fluorescence hydrogel	[226]
	SOD, CAT	MSCO	Rough-surfaced nanosheets	Collagen deposition Proangiogenic Antibacterial	Composite hydrogel	[228]
ATP-Responsive	OXD	Ce-UiO-66-NO_2_	Isoreticular-structured nanocrystals	Antibacterial	DMF solution	[232]
ROS-Responsive	SOD	AuNCs@PBA-Sa	Ultrasmall nanoclusters	Immunomodulation Anti–inflammatory Pro-regeneration	Self-adaptive hydrogels	[237]
Microenvironment-Responsive	POD	Apt-PtNZs	Star-shaped nanoparticles	Antibacterial	Nanocapsule	[113]
pH and NIR light-responsive	Alkaline: CAT Acidic: POD	CuP	Nanosheets	Antibacterial Pro-regeneration	Alginate hydrogel	[246]
ROS and glucose-responsive	SOD, CAT	CHA@GOx	Spherical nanoparticles	Re-epithelialisation Collagen deposition Proangiogenic Fibroblast migration	Aqueous solution	[81]
pH and ROS-responsive	POD	FEMI	2D anisotropic nanosheets	Regulating blood sugar Anti–inflammatory Proangiogenic Re-epithelialisation Antibacterial	Injectable multifunctional hydrogel	[155]
NIR light and US-responsive	OXD	PtRu/C_3_N_5_	Ultrasmall nano-alloy-decorated porous nanosheets	Antibacterial Anti–inflammatory	Hyaluronic acid microneedles	[201]
Temperature, pH, and NIR Light-Responsive	SOD, CAT	CMCS/PNIPAM	Nanosheets	Alleviated oxidative stress Collagen deposition Proangiogenic Antibacterial	Self-adaptive dressings	[101]

### Biosafety and clinical translation

When nanozyme systems are administered into living organisms, their biological fates should be systematically investigated. The biosafety of nanozymes presents an inescapable challenge before their translation into clinical applications. In contrast to natural enzymes, nanomaterial-based biomedicines raise common concerns about their biocompatibility and biodegradability. The metabolic fate of nanozymes is a crucial concern that requires attention. The clinical application of nanozymes is still hampered by their in vivo toxicity [259].

Currently, systemic injections of nanozymes can harm healthy tissues. This is particularly true for metal-based nanozymes, whose toxicity mainly arises from ROS production due to their enzyme-mimicking activity. In some cases, the metal ions used in their synthesis are not essential for organism function, and certain components may be toxic, compromising therapeutic efficacy [39]. Due to their small size and high surface activity, nanozymes can easily penetrate cell membranes and interact with cellular genetic material. After entering the cell, metal ion-carrying nanozymes may induce breaks in chromosomes or DNA due to oxidative stress or inflammation [260]. The toxicity of metal-based nanozymes is primarily dependent on the metal species used to construct them [39]. Degradation products may be produced through the biodegradation or metabolic processes of nanozymes, and these products have different properties or biological effects from the original nanozymes. It is important to study the composition and biological activity of these degradation products to understand their long-term effects. The fate of nanozymes in the body involves various complex interactions with biological systems, including biodegradation, clearance, metabolism, excretion, toxicity, immunogenicity, and the formation of degradation products. Therefore, comprehensive studies are necessary to elucidate these processes and ensure the safety and efficacy of nanozymes in long-term biomedical applications. For instance, excessive copper or iron in normal tissue/cells may lead to a Fenton or Fenton-like reaction that can cause damage to biomacromolecules and nucleic acids [261]. Therefore, it is crucial to evaluate the pharmacokinetics of nanozymes to ensure their biosafety and biocompatibility. Only a limited amount of research has been carried out on the potentially toxic effects of nanozymes, such as genotoxicity and reproductive toxicity. The bioavailability of functional nanozymes and their impacts on whole-body metabolism should be explored. In conclusion, a comprehensive investigation into the metabolism and biosafety of nanozymes is essential for promoting their progress to the clinical trial stage.

The interactions between nanozymes and biological systems should be carefully examined, and efforts should focus on synthesizing nanozymes with both high safety profiles and enzyme catalytic activity through structural optimization and surface modification [262]. The use of carriers for drug delivery can provide more precise release of nanozymes at wound sites. Synthesis and modification relying on biomolecules (e.g., PEG and bovine serum albumin) are prospective strategies for obtaining nanozymes with high biocompatibility [263]. Surface modification is a promising strategy to overcome the limitations of nanozymes because their tunable properties provide opportunities to design biosafe agents [264]. In recent years, cell membrane coating technology has emerged as a novel bioinspired method for modifying nanozymes, thanks to their convenient functional properties and excellent biological features [265]. It is necessary to develop more convenient and controllable nanozyme-delivery routes, enhance the delivery efficiency and intrinsic activity of nanosystems, and improve the bioavailability of nanodrugs. Furthermore, using various carriers such as hydrogels, protein nanocages, and liposomes can prevent the decrease in enzyme-like catalytic activity induced by the direct surface modification of nanozymes. This approach also enhances biocompatibility, controls drug release, and improves targeting. However, studies are still in their early stages, and many significant problems remain to be resolved. Additionally, the choice of support materials significantly influences the therapeutic outcomes, bioavailability, clearance dynamics, and systemic toxicity of nanozymes.

For successful and effective wound therapy, the toxicity of nanozymes should be reduced while maintaining their enzyme activity at the treatment site. Moreover, it is important to note that regulating the activity of nanozymes is currently challenging. Oxidative processes can spontaneously initiate in an aqueous environment, potentially causing harmful side effects on healthy tissues. Consequently, it is highly desirable to simultaneously enhance and precisely control the activity of nanozymes specific to lesion areas [205]. The enzymatic response is expected to possess a high level of damage site specificity, thus guaranteeing its biocompatibility and therapeutic specificity. In particular, under effective treatment conditions, the dosage of nanozymes can be reasonably controlled to minimize toxic side effects and promote efficient wound healing.

### Specificity

Improving the selectivity and specificity of nanozymes is crucial for their further optimization and application. Nanozymes with targeting capabilities are highly desirable. Although nanozymes possess numerous distinctive advantages, they still lack the ability to target the lesion site, which unavoidably causes certain damage to normal cells. To address this issue, modifying targeted molecules on the surface of nanozymes is a prevalent solution. Nanozymes can be employed for precise drug delivery through the incorporation of specific ligands onto their surfaces, allowing them to selectively bind to disease-related biomarkers [115]. This targeted strategy facilitates the direct delivery of therapeutic agents to the affected tissues, reduces off-target effects, and enhances the efficacy of drug treatments for metabolic diseases. For instance, P/N-doped porous carbon nanozymes preloaded with indole-3-acetic acid were modified with folic acid to confer tumor-targeting ability on the nanozyme [266]. A novel strategy for noninvasive active-targeting therapy in ischemic stroke entails the utilization of a mesoporous Prussian blue nanozyme coated with a neutrophil-like cell membrane. This inventive approach augments the targeted delivery of therapeutic agents to the injured brain by taking advantage of the intrinsic relationship between inflamed brain microvascular endothelial cells and neutrophils after a stroke [267]. To enhance biocompatibility and promote cellular uptake, Pt nanozymes were coated with triphenylphosphonium-conjugated liposomes. This coating allowed the nanozymes to bypass the lysosomal barrier, penetrate the cell membrane, and specifically target mitochondria for effective scavenging of mitochondrial O_2_^•−^ and to relieve hypoxia [268].

### Limited understanding of mechanism

The mechanisms of the majority of responsive nanozymes remain ambiguous. The specific structures of nanozymes exhibit a strong correlation with their catalytic profiles. A comprehensive understanding of the structure-activity relationship guarantees a superior design and utilization of stimuli-responsive nanozymes, and a more profound understanding of the specific mechanisms of stimuli-response is beneficial for the rational design of ideal nanozymes and the precise regulation of their catalytic activities [115]. The catalytic profiles of diverse nanozymes ought to be thoroughly investigated. Specific mechanisms need to be comprehensively comprehended to enable greater control over the reactions. For instance, numerous nanozymes can accept a wide variety of substrates and thus exhibit multiple enzyme-mimicking activities [269]. Although such a property may be appealing, it will also lead to concerns regarding catalytic specificity and substrate selectivity. Rational design and surface chemistry of nanozymes are essential for determining their catalytic performance and, consequently, their biomedical applications. A number of studies have reported discrepant mechanisms of nanozyme catalysis, like those of nanoceria [262]. Such evidence underlines the difficulties in establishing standard catalytic profiles of nanozymes. Experimental and computational studies can significantly contribute to the nanozyme design process and mechanism investigations. Furthermore, substituting time-consuming verification experiments with efficient simulation or prediction has become a prevalent trend lately. Machine learning or other artificial intelligence technologies may hold broad prospects in the design of novel stimuli-responsive nanozymes on account of their efficient screening and rational prediction [270].

The ultimate goal of biomedicine is clinical translation, which requires in vivo exploration of the therapeutic mechanisms and long-term effects of nanozymes. Research on nanozyme technology has primarily focused on phenotypic effects (e.g., anti-inflammatory, antibacterial, and signaling pathway regulation), while studies on gene therapy, cell therapy, and growth factor therapy remain limited [271]. In-depth studies of specific mechanisms, including action sites and molecular interaction targets, are crucial for optimizing nanozyme design and guiding clinical drug delivery. External modifications are likely to modify the physicochemical properties and wound healing pathways of nanozymes [269]. Consequently, it is essential to use computer and machine-learning technologies to model the kinetics of nanozyme catalysis and examine the energetic changes during reactions. This approach will help reveal the intrinsic catalytic mechanism and clarify its constitutive relationships [270]. Currently, most research is based on rodents for in vivo studies of nanozymes, which differs significantly from human biological systems [271]. For a more reliable prediction of the efficacy and toxicity of nanozymes, in vivo experiments on large mammals will be required in the future. Currently, nanozymes are in the early stages of development, with uncertainties in their specific mechanisms posing challenges to their immediate clinical application. Significant concerns remain regarding chronic toxicity, genotoxicity, carcinogenicity, and therapeutic efficacy in large animal models. These issues pose hurdles for Food and Drug Administration approval. From a commercial perspective, considerations such as Good Manufacturing Practice compliance and shelf stability are vital and warrant further investigation. Increased research efforts aimed at understanding nanozymes’ mechanisms and demonstrating their enhanced therapeutic effects hold promise for a bright future in their development and application.

### Reproducibility and scalability

Although the substitution of protein enzymes with nanozymes in industrial and medical applications is approaching, further efforts are still required. Improving the batch-to-batch reproducibility of nanozymes remains vital for their industrial or clinical translation. Although nanozymes possess substantial potential for diverse applications, certain limitations regarding their reproducibility and scalability demand tackling. One obstacle lies in guaranteeing the reproducibility of nanozyme synthesis and characterization. Multiple factors, such as the synthesis method, reaction parameters, and surface modifications, can influence the properties and catalytic activity of nanozymes [272]. Minor fluctuations in these parameters may result in substantial disparities in nanozyme performance. For example, He et al. found that small, low-crystallinity Cys-PB-NPs boost POD-like activity by increasing crystal vacancy defects. In addition, this size-defect synergy primarily drives POD-like activity, resulting in greater structural sensitivity compared to SOD- and CAT-like activities [260]. Bao et al. explored how nanoparticle morphology affects nanozyme performance by synthesizing cerium oxide nanoparticles in three shapes: nanocone, nanopolyhedron, and nanoflower. The nanoflower structures exhibited the highest enzymatic activity due to their large specific surface area and optimal Ce^4+^/Ce^3+^ ratio. This combination enhanced their free radical scavenging efficiency [273]. Consequently, it is crucial to set up standardized protocols and characterization techniques for ensuring the reproducibility of nanozyme synthesis and evaluation. Another constraint pertains to the scalability of nanozyme production. A great number of nanozymes are synthesized via complex and time-consuming methods, which might not be readily scalable for large-scale production [274]. Moreover, the synthesis of certain nanozymes necessitates using costly or toxic reagents, further impeding their scalability.

## Conclusion

The treatment of wound healing still poses a challenging task in clinical practice, as current approaches produce only limited therapeutic effects. Responsive nanozymes have been extensively utilized in wound treatment on account of their low cost, facile functionalization, biological inertness, and precise adjustability. This review presents the switch of nanozyme activities and the release of nanozymes under endogenous or exogenous stimuli, such as pH value, overexpressed H_2_O_2_, heat, US, and light irradiation in wound healing. Responsive nanozymes are anticipated to concurrently modulate wound healing via multiple pathways (including blood-glucose reduction, hypoxia amelioration, oxidative-stress mitigation, and bacterial-infection eradication) at all stages and offer a balanced environment during the entire wound healing process, thus diminishing potential side effects. The stimuli-switchable activity and stimuli-triggered release are highly significant for in vivo applications, aiming to maximize the nanozymes’ therapeutic effects while minimizing damage to normal tissues. The excellent designability and versatility of stimuli-responsive nanozymes provide boundless possibilities for the future development of responsive nanozymes based on a comprehensive understanding of the disease microenvironment and the structure–activity relationship of materials.

## Data Availability

No datasets were generated or analysed during the current study.

## Abbreviations

*S. aureus*: *Staphylococcus aureus*
DFU: diabetic foot ulcers
NPs: nanoparticles
MOFs: metal organic frameworks
ROS: reactive oxygen species
SOD: superoxide dismutase
CAT: catalase
GSH-Px: glutathione peroxidase
POD: peroxidase
OXD: oxidase
H_2_O_2_: hydrogen peroxide
GSH: glutathione
US: ultrasound
O_2_: oxygen
•OH: hydroxyl radical
O_2_^•−^: superoxide anion
NOS: nitric oxide synthase
NIR: near-infrared
CoCNI: halogen-doped cobalt cyanide
NO: nitric oxide
ZLGs: zwitterionic lysine glycopolymers
GOx: glucose oxidase
2D: two-dimensional
CDT: chemodynamic therapy
MoS_2_: molybdenum disulfide
HRP: horseradish peroxidase
WN: tungsten nitride
VEGF: vascular endothelial growth factor
HIF-1α: hypoxia-inducible factor 1α
MnO_2_: manganese dioxide
PTT: photothermal therapy
GQDs: Graphene quantum dots
NADH: nicotinamide adenine dinucleotide
SDT: sonodynamic therapy
PNA: platinum nanozyme
GA: gallic acid
CG: cationic guar gum
ATP: adenosine triphosphate
